# Single-Cell RNA Sequencing, Cell Communication, and Network Pharmacology Reveal the Potential Mechanism of *Senecio scandens* Buch.-Ham in Hepatocellular Carcinoma Inhibition

**DOI:** 10.3390/ph17121707

**Published:** 2024-12-18

**Authors:** Jiayi Jiang, Haitao Wu, Xikun Jiang, Qing Ou, Zhanpeng Gan, Fangfang Han, Yongming Cai

**Affiliations:** 1 School of Medical Information and Engineering, Guangdong Pharmaceutical University, Guangzhou 510006, China; jiangjiayixinxiang@163.com (J.J.); t810134589@163.com (H.W.); jiang_xikun99@163.com (X.J.); oq1007918@163.com (Q.O.); 2122241020@gdpu.edu.cn (Z.G.); 2 NMPA Key Laboratory for Technology Research and Evaluation of Pharmacovigilance, Guangzhou 510300, China; 3 Guangdong Provincial Traditional Chinese Medicine Precision Medicine Big Data Engineering Technology Research Center, Guangzhou 510006, China

**Keywords:** *Senecio Scandens* Buch.-Ham, hepatocellular carcinoma, network pharmacology, *SRC*, *FOS*

## Abstract

Background: Hepatocellular carcinoma (HCC), a prevalent form of primary liver malignancy, arises from liver-specific hepatocytes. *Senecio scandens* Buch.-Ham(Climbing senecio) is a bitter-tasting plant of the Compositae family with anti-tumor properties. This study aims to identify the molecular targets and pathways through which Climbing senecio regulates HCC. Methods: Active ingredients of Climbing senecio were collected from four online databases and mapped to relevant target databases to obtain predicted targets. After recognizing the key pathways through which Climbing senecio acts in HCC. Gene expression data from GSE54238 Underwent differential expression and weighted gene correlation network analyses to identify HCC-related genes. The “Climbing senecio-Hepatocellular Carcinoma Targets” network was constructed using Cytoscape 3.10.1 software, followed by topology analysis to identify core genes. The expression and distribution of key targets were evaluated, and the differential expression of each key target between normal and diseased samples was calculated. Moreover, single-cell data from the Gene Expression Omnibus (GSE202642) were used to assess the distribution of Climbing senecio’s bioactive targets within major HCC clusters. An intersection analysis of these clusters with pharmacological targets and HCC-related genes identified Climbing senecio’s primary targets for this disease. Cell communication, receiver operating characteristic (ROC)analysis, survival analysis, immune filtration analysis, and molecular docking studies were conducted for detailed characterization. Results: Eleven components of Climbing senecio were identified, along with 520 relevant targets, 300 differentially expressed genes, and 3765 co-expression module genes associated with HCC. *AKR1B1*, *CA2*, *FOS*, *CXCL2*, *SRC*, *ABCC1*, and *PLIN1* were identified within the intersection of HCC-related genes and Climbing senecio targets. TGFβ, IL-1, VEGF, and CXCL were identified as significant factors in the onset and progression of HCC. These findings underscore the anti-HCC potential and mode of action of Climbing senecio, providing insights into multi-targeted treatment approaches for HCC. Conclusions: This study revealed that Climbing senecio may target multiple pathways and genes in the process of regulating HCC and exert potential drug effects through a multi-target mechanism, which provides a new idea for the treatment of HCC. However, the research is predicated on network database analysis and bioinformatics, offering insights into HCC therapeutic potential while emphasizing the need for further validation.

## 1. Introduction

Hepatocellular carcinoma (HCC), a prevalent form of primary liver malignancy, arises from liver-specific hepatocytes. The incidence of liver cancer varies significantly worldwide, particularly in East Asia and Africa [1]. Encompassing a range of elements, the development of hepatocellular carcinoma is influenced by chronic infections with hepatitis B(HBV) or hepatitis C(HBC), along with liver cirrhosis, alcohol overconsumption, and the presence of nonalcoholic fatty liver disease [2]. Common symptoms in liver cancer patients include epigastric pain, loss of appetite, weight loss, jaundice, and ascites. These symptoms may differ among individuals and overlap with those of other liver diseases, complicating early diagnosis [3].

Treatment options for HCC encompass surgery, localized ablation, radiation therapy, chemotherapy, and therapies aimed at specific molecular targets [4]. In suitable patients, liver transplantation offers a curative option that not only removes tumors but also addresses liver failure due to cirrhosis [5]. Despite advancements in treatment, outcomes for liver cancer are strongly affected by the cancer stage at diagnosis and the overall health of the patient. Thus, early detection and intervention are critical for improving treatment efficacy and survival rates. Traditional Chinese medicine (TCM) has contributed significantly to human health worldwide and plays an important role in treating tumors [6]. Increasing clinical evidence indicates that many malignancies can be prevented and treated with TCM. Recent trials have also shown that TCM effectively reduces the prevalence of HCC and improves patient prognosis [7,8]. Therefore, the development of novel therapeutic targets and approaches remains essential in the management of HCC.

*Senecio scandens* Buch.-Ham (Climbing senecio) is a Compositae family plant known for its bitter taste and cold nature [9]. Its pharmacological effects include antibacterial, anti-inflammatory, anti-tumor, and antioxidant properties, and it is used to enhance vision, promote diuresis, and eliminate heat and toxins [10]. The anti-tumor properties of Climbing senecio are attributed to its rich composition of bioactive compounds, including sesquiterpenes, phenolic acids, flavonoids, and alkaloids [11]. Of these, flavonoids such as quercetin and kaempferol, as well as pyrrolizidine alkaloids, are particularly recognized for their potent anticancer activities [12]. These compounds mediate their effects through various mechanisms, including induction of apoptosis, tumor cell proliferation inhibition, angiogenesis suppression, and immunomodulation [13,14]. Despite these promising attributes, the pharmacological complexity of Climbing senecio poses significant challenges in isolating and identifying the specific active components and molecular targets responsible for its effects on HCC. The mechanism by which Climbing senecio affects liver cancer remains unclear [15].

Network pharmacology and bioinformatics are effective strategies for exploring potential mechanisms in cancer development and drug action [16]. These methods help identify key genes, proteins, or metabolites that may play a crucial role in Climbing senecio’s regulation of liver cancer. By comparing gene expression data and conducting functional enrichment analysis, the regulatory effects of Climbing senecio on liver gene expression can be elucidated, revealing potential toxicological mechanisms. Analysis of intercellular communication within annotated data enables the exploration of relationships between cell types and subsets. Molecular docking further verifies the affinity between active components and targets, providing accurate guidance for clinical application. This study aims to investigate the regulatory effects of Climbing senecio on liver cancer using network pharmacology, genome and transcriptome data analysis, and molecular docking.

## 2. Results

### 2.1. General Components and Targets of Climbing senecio

**Figure 1:**
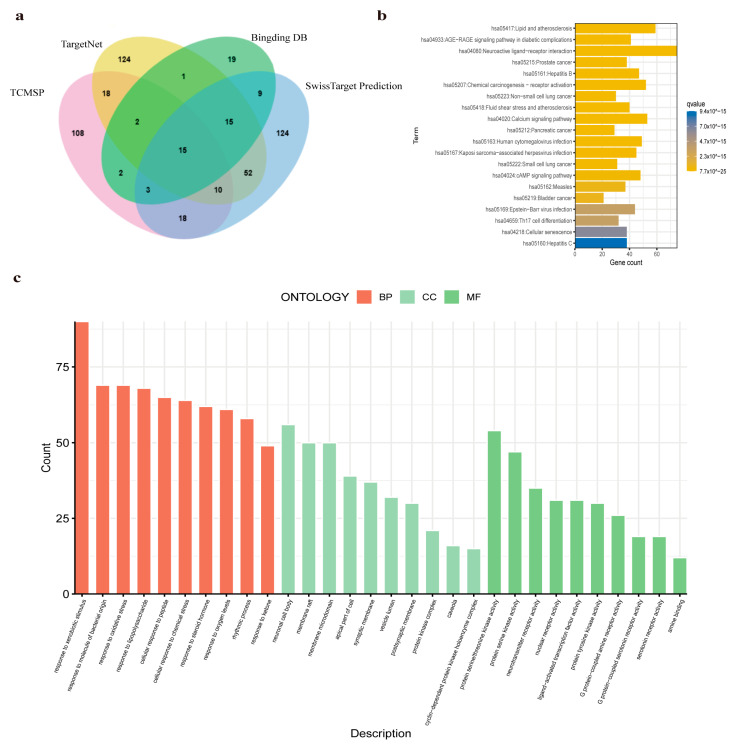
Identification and analysis of targets for *Senecio scandens* Buch.-Ham (Climbing senecio). (**a**) Venn diagram of Climbing senecio targets across TCMSP, TargetNet, Binding DB, and SwissTargetPrediction. (**b**) Enrichment analysis of Climbing senecio targets using the Kyoto Encyclopedia of Genes and Genomes (KEGG) pathway database. (**c**) Comprehensive Gene Ontology (GO) enrichment analysis for Climbing senecio, including categories of biological processes (BP), cellular components (CC), and molecular functions (MF).

The components of Climbing senecio were collected from four databases, including 50 types from TCMBank (https://tcmbank.cn/, accessed on 15 March 2024), 47 types from TCM-ID (https://www.bidd.group/TCMID/, accessed on 15 March 2024), 57 types from Herb (http://herb.ac.cn/, accessed on 15 March 2024), and 19 types from SymMap (http://www.symmap.org/, accessed on 15 March 2024). After merging and de-duplicating these data, 99 compounds of Climbing senecio were retained. From the TCMSP database (https://www.tcmsp-e.com/#/database, accessed on 15 March 2024), 12 candidate active components were identified (Supplementary Table S1). Moreover, 250 and 176 related targets of Climbing senecio were identified from the SwissTargetPrediction (http://www.swisstargetprediction.ch/, accessed on 15 March 2024) and TCMSP databases, respectively. Through the BindingDB (http://www.bindingdb.org/bind, accessed on 15 March 2024) and TargetNet (http://targetnet.scbdd.com/, accessed on 15 March 2024) databases, 66 and 236 additional potential pharmacological targets were identified, respectively (Figure 1a and Supplementary Table S2). After merging and de-duplicating, a total of 520 targets associated with drugs were chosen for enrichment analysis using Gene Ontology (GO) and the Kyoto Encyclopedia of Genes and Genomes (KEGG).

The GO biological processes associated with these targets are primarily concentrated in response to xenobiological processes, response to molecules of bacterial origin, and response to oxidative stress. The cell components identified in GO analysis mainly include neuronal cell body, membrane raft, and membrane microdomain. Key molecular functions of these targets are enriched in protein serine/threonine kinase activity, neurotransmitter receptor activity, and nuclear receptor activity (Figure 1b). The KEGG pathway analysis of the pharmacological targets reveals several key pathways, including the neuroactive ligand–receptor interaction, the AGE-RAGE signaling pathway implicated in diabetic complications, and pathways associated with lipid metabolism and atherosclerosis. These results collectively indicate that Climbing senecio potentially modulates the activity of protein serine/threonine kinases (Figure 1c).

### 2.2. Target Genes in HCC

**Figure 2:**
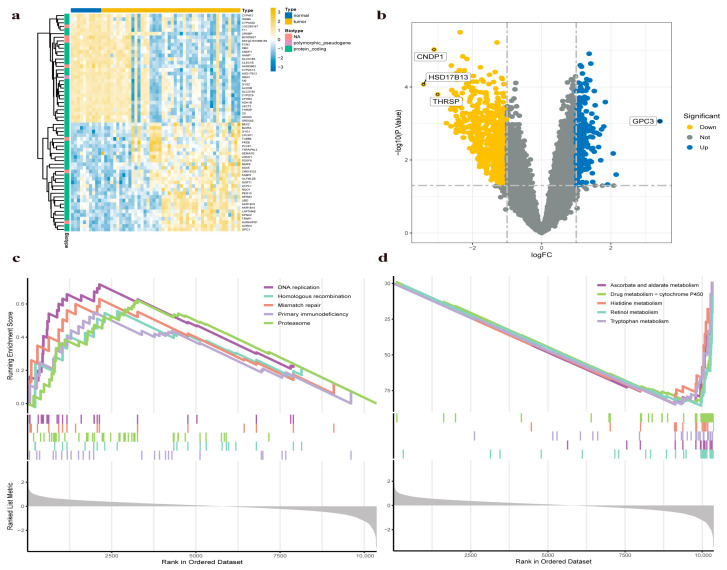
Differentially expressed genes (DEGs) in the GSE54238 dataset. (**a**) The heatmap displaying the expression profiles of DEGs. (**b**) Volcano plot illustrating the distribution of DEGs. (**c**,**d**) Gene set enrichment analysis (GSEA) based on KEGG pathways.

A total of 56 liver disease-related samples from GSE54238. The dataset GSE54238, sourced from the GEO database at https://www.ncbi.nlm.nih.gov/geo/query/acc.cgi?acc=GSE54238, accessed on 18 March 2024, comprises a collection of samples: 10 from normal livers (NL), 10 from chronically infected livers (IL), 10 from cystic hepatic livers (CL), 13 from early-stage hepatocellular carcinoma (EHCC), and 13 from advanced-stage hepatocellular carcinoma (AHCC). Ten normal samples were labeled “normal”, while 46 disease samples were labeled “tumor” in the control group. The original sequencing data were normalized and differentially expressed genes (DEGs) between the control groups (normal and tumor tissues) were identified. Of these genes, 223 were downregulated, while 77 were upregulated (Supplementary Table S3). A volcano plot highlights the top four DEGs and a heatmap displays 60 significant DEGs (Figure 2a,b). Gene set enrichment analysis (GSEA) was conducted to evaluate the enrichment of biological pathways in the disease group compared to the normal group. The results indicate enrichment of DNA replication, homologous recombination, mismatch repair, primary immunodeficiency, and proteasome pathways in the disease group (Figure 2c). In contrast, ascorbic acid and aldolase metabolism, drug metabolism-cytochrome P450, histidine metabolism, retinol metabolism, and tryptophan metabolism were inhibited (Figure 2d).

### 2.3. Weighted Gene Co-Expression Network Analysis (WGCNA) Confirms Key Modules

**Figure 3:**
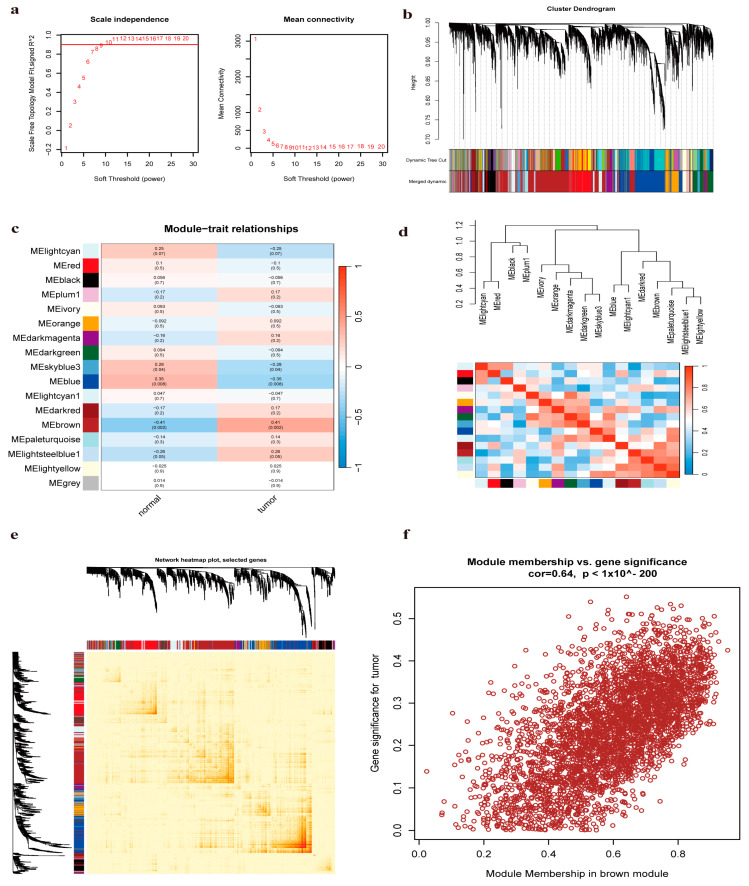
Weighted gene co-expression network analysis (WGCNA) of enrichment values. (**a**) Soft threshold selection. (**b**) WGCNA cluster dendrogram. (**c**) Gene module separation and cluster dendrogram in WGCNA, with different colors representing different modules. (**d**) Inter-module correlation. (**e**) Module-trait relationship analysis diagram for 17 modules. (**f**) Relationship between gene significance and brown module memberships.

We utilized microarray data from the GSE54238. Outlier detection confirmed the absence of significant outliers in the data. The scale-free topology fit index reached a value of 0.9, and a soft-threshold power of 8 was determined to be optimal for ensuring data connectivity (Figure 3a). Correlation and topological overlap matrices were constructed between genes. A clustering tree was generated with dynamic tree cutting and merged dynamic modules, producing a co-expression network (Figure 3b). Seventeen modules were finally identified from the clustering results (Figure 3c). Correlation coefficients between each module and HCC-associated phenotypes were calculated (Figure 3d). The MEbrown module was the most relevant in the disease group (correlation coefficient = 0.41, *p* = 0.002). Figure 3e illustrates a correlation heatmap for the identified modules, and Figure 3f depicts a scatter plot comparing gene significance (GS) to module membership (MM), demonstrating a robust correlation specifically within the MEbrown module, with a correlation coefficient (R) of 0.64 and a *p*-value less than 1 × 10^−200^. Collectively, the MEbrown module appears to best represent the pathological changes associated with HCC.

### 2.4. Identification of Key Targets

**Figure 4:**
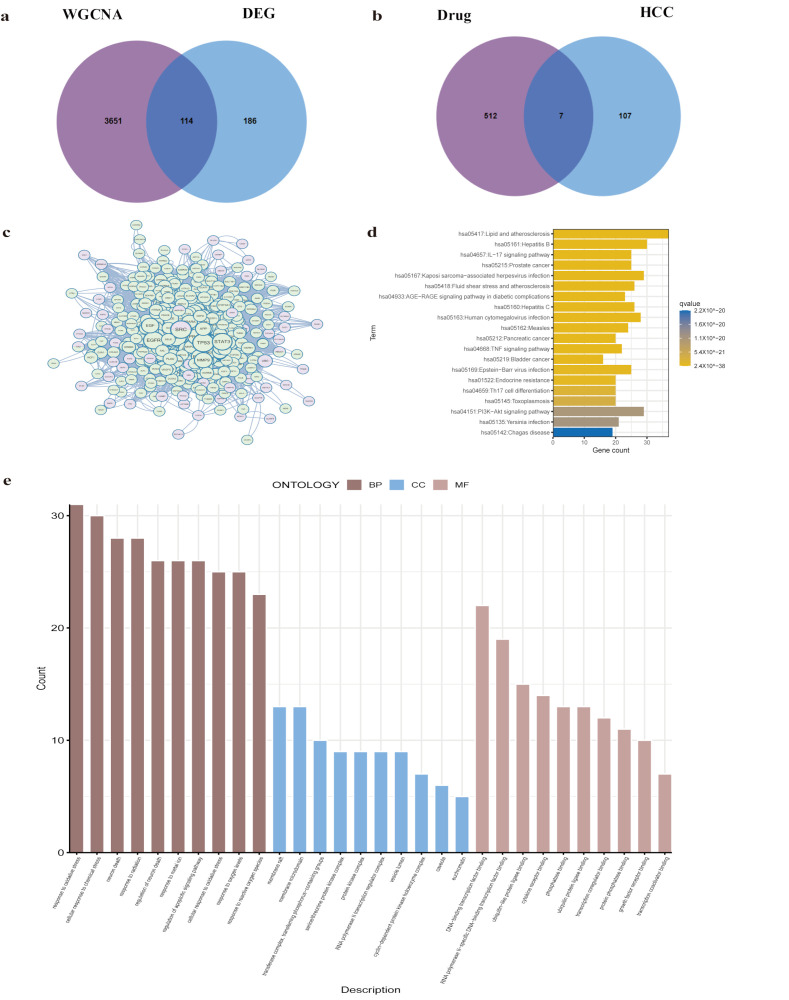
Key target identification and functional analysis. (**a**) The intersection of Climbing senecio-related genes with DEGs and WGCNA brown module genes. (**b**) Intersection of drug targets with Climbing senecio-related genes. (**c**) The Climbing senecio–HCC protein interaction network generated in Cytoscape3.10.1 showing Climbing senecio-related genes and drug targets. Green and light pink indicate both Climbing senecio-related genes and drug targets; light green indicates drug targets; light pink indicates Climbing senecio-related genes. (**d**) KEGG pathway analysis of key genes. (**e**) GO analysis of the primary cluster.

A total of 114 genes associated with HCC were identified by comparing the DEGs with genes in the MEbrown module (Figure 4a and Supplementary Table S3). Seven key genes—*AKR1B1*, *CA2*, *FOS*, *CXCL2*, *SRC*, *ABCC1*, and *PLIN1*—were found at the intersection between drug targets and HCC-related genes (Figure 4b). A protein–protein interaction (PPI) network for genes linked to both drugs and HCC was constructed using the STRING online database, which was then analyzed and visually presented using Cytoscape 3.10.1 software (Figure 4c). The 627 targets associated with Climbing senecio and HCC were uploaded to the STRING database, and the PPI network was constructed using the species selection “*Homo sapiens*”. The resulting Climbing senecio–HCC protein interaction network comprised 610 effective nodes and 12,812 edges, with an average node degree of 42, a local clustering coefficient of 0.498, and a PPI enrichment *p* value of <1.0 × 10^−16^. GO analysis of these important targets reveals that oxidative stress increases biological processes, including cellular response to chemical stress, neuron apoptotic process, and other stress-related responses. The biological processes show that key clusters are mainly concentrated in response to oxidative stress, cellular response to chemical stress, and neuron death. Molecular function analysis highlights the importance of DNA-binding transcription factor binding, RNA polymerase II-specific DNA-binding transcription factor binding, and ubiquitin-like protein ligase binding (Figure 4e). KEGG enrichment analysis shows that these significant targets are mainly involved in pathways related to hepatitis B, lipid and atherosclerosis, and the PI3K-Akt signaling pathway (Figure 4d).

### 2.5. Single-Cell RNA Sequencing Data Analysis

**Figure 5:**
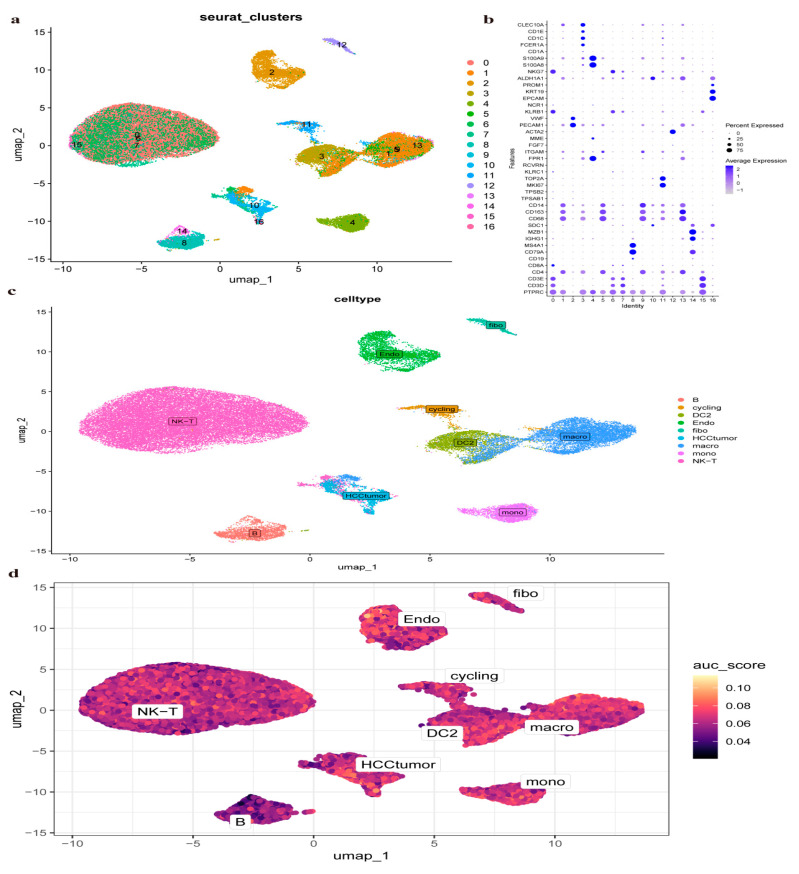
Single-cell overview in HCC. (**a**) Unified clustering into 17 clusters. (**b**) Bubble charts at each gene table level. (**c**) Identification of nine clusters. (**d**) Proposed pathway of Climbing senecio’s action on HCC.

Individual cell data were extracted from the GSE202642 (https://www.ncbi.nlm.nih.gov/geo/query/acc.cgi?acc=GSE202642, accessed on 6 April 2024). To ensure high-quality analysis, cells were filtered according to specific criteria: the number of genes (nFeature_RNA) was set between 300 and 7000, mitochondrial gene proportion (mt_percent) was kept below 10%, hemoglobin gene proportion (HB_percent) was below 3%, and the total UMI count (nCount_RNA) was above 1000 and below the 97th percentile of all UMI counts (Supplementary Figure S1a). The “Harmony” package was used to eliminate batch effects (Supplementary Figure S1b). A resolution of 0.2 was set for the clustering tree, with a principal component value of 25. The uniform manifold approximation and projection (UMAP) results identified 17 distinct cell clusters (Figure 5a).

A bubble chart (Figure 5b) visualizes the distribution of gene types within each cluster. The CellMarker database was utilized to identify and analyze specific cell markers, and nine cell types were annotated: B cells, NK/T cells, stromal cells (endo), HCC tumor cells, macrophages (Mac), dendritic cells (DC), monocytes (Mono), proliferating cells (cycling), and fibroblasts (fibo) (Figure 5c). The distribution of drug targets was assessed using AUCell function score analysis, showing that Climbing senecio primarily affects dendritic cells, stromal cells, and HCC cell clusters (Figure 5d).

### 2.6. The Expression and Distribution of Key Targets

**Figure 6:**
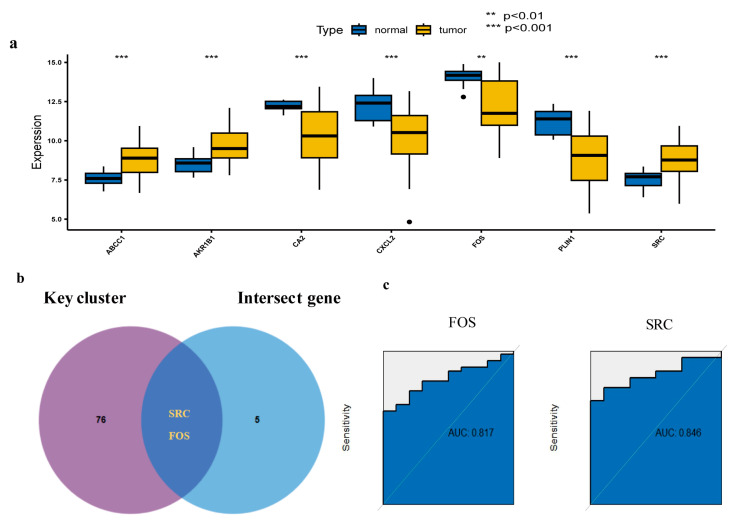
Expression and distribution of the key cluster with receiver operating characteristic (ROC) curve analysis. (**a**) A boxplot depicting the differential expression of pivotal genes between normal and control tissues within the GSE54238. (**b**) The crucial targets are determined by the overlap of key clusters with genes associated with Climbing senecio and targets linked to HCC. (**c**) ROC curve analysis of three crucial targets.

The differential expression and distribution of each key target between normal and diseased samples were analyzed, with results presented in a box plot (Figure 6a). The key cluster with a score of 61.143 was obtained by molecular complex detection (MCODE) analysis, which contained 78 key genes. Two significant targets were identified by comparing crossover genes with critical targets at the intersection of pharmacological targets and HCC-related genes (Figure 6b). ROC curve analysis demonstrates strong robustness for these two targets in HCC, with an area under the ROC curve (AUC) > 0.8 (Figure 6c). Among the key targets, *SRC* expression is upregulated, whereas *FOS* expression is downregulated compared to normal samples. The survival analysis revealed a substantial correlation between the levels of expression of the two specific genes (*SRC*, *FOS*) and the prognosis of patients with HCC, as depicted in Supplementary Figure S2.

### 2.7. Cell Communication Analysis 

**Figure 7:**
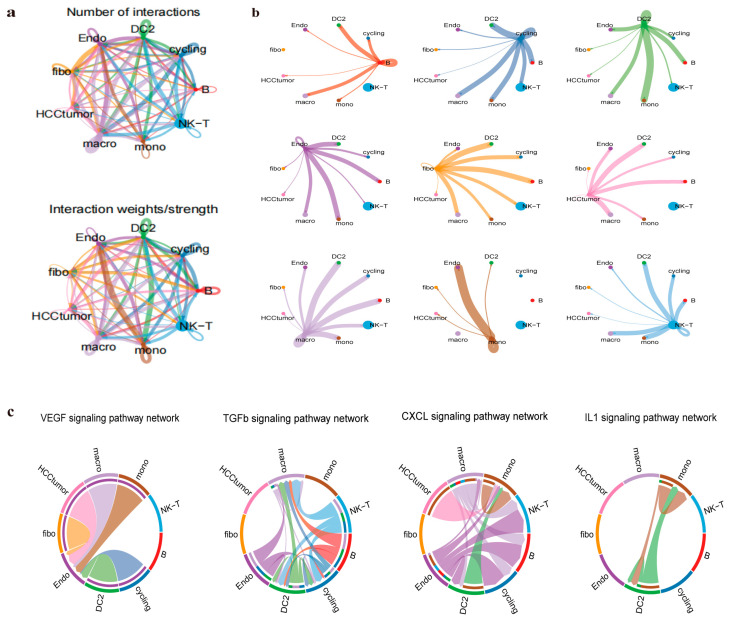
Key pathways in intercellular communication analysis. (**a**) Cellular interaction network. (**b**) Interaction between cell types. (**c**) Network of TGF-β, IL-1, CXCL, and VEGF signaling pathways.

In investigating cell communication within HCC, complex interactions were observed among eight cell types and HCC cells. The communication interaction weights between all cell types are shown in Figure 7a,b. Key signaling molecules, including transforming growth factor beta (TGFβ) related to *SRC*, interleukin 1 (IL-1) associated with *FOS*, CXC chemokine (*CXCL2*), and vascular endothelial growth factor (VEGF), were highlighted due to their critical roles in tumor initiation, progression, and deterioration. The Src kinase, encoded by the *SRC* gene, is an essential component of the TGFβ signaling pathway and influences tumor cell invasion and metastasis [17] (Figure 7c). The *FOS* gene encodes a protein that is a component of the AP-1 transcription factor complex, which is active in IL-1 signal transduction. IL-1 may promote the occurrence and development of tumors in HCC by inducing chronic inflammation. *CXCL2*, a member of the CXCL chemokine family, plays a significant role in inflammatory responses, closely associating inflammation and cancer. In HCC, *CXCL2* and its family members can shape the tumor microenvironment. VEGF signaling may induce *CXCL2* secretion by tumor-associated macrophages (TAMs) or other cell types, attracting more monocytes into the tumor microenvironment through immune cell recruitment at the tumor site. The interactions among these genes and signaling pathways affect HCC progression, suggesting that Climbing senecio may regulate HCC development by targeting *FOS*, *CXCL2*, and *SRC*.

### 2.8. Immune Infiltration Analysis

**Figure 8:**
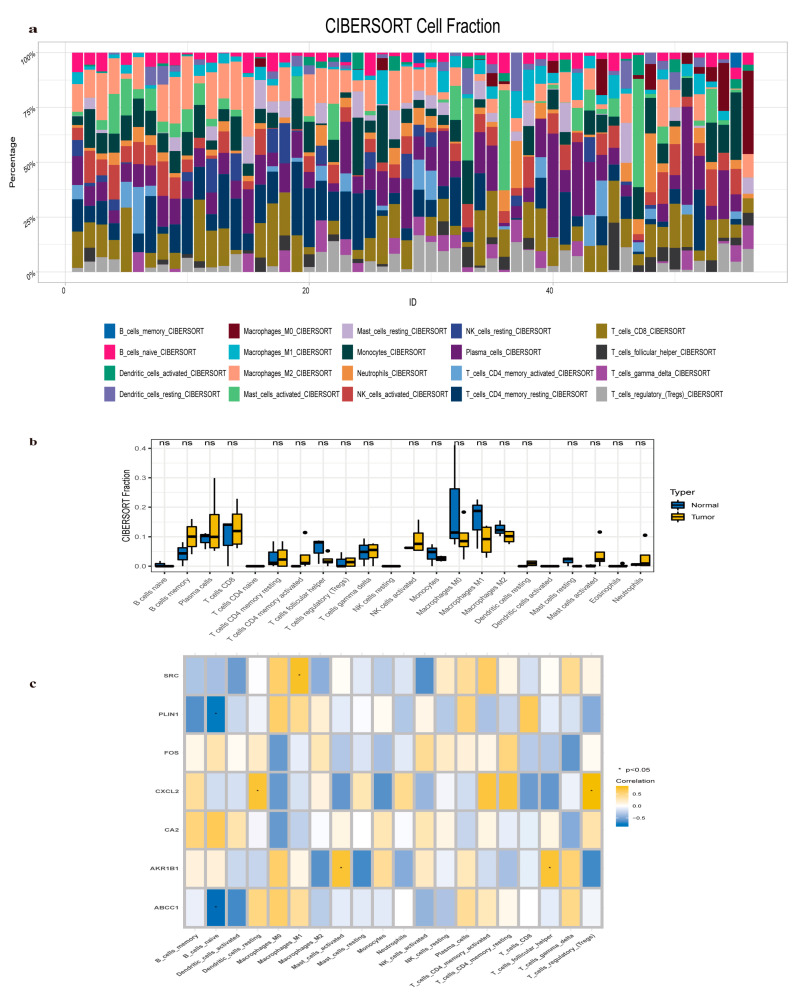
Immune filtration analysis of *AKR1B1*, *CA2*, *FOS*, *CXCL2*, *SRC*, *ABCC1*, and *PLIN1*. (**a**) Stacked column diagram of 20 types of immune cell infiltration in the GSE54238 dataset. (**b**) A box diagram illustrating the variation in infiltration levels of different immune cell types between diseased and normal samples. The “ns” (not significant) means there is no statistically significant difference. (**c**) A heatmap depicting the correlations between immune cell infiltration and the expression levels of *AKR1B1*, *CA2*, *FOS*, *CXCl2*, *SRC*, *ABCC1*, and *PLIN1*.

Immune infiltration was evaluated using CIBERSORT to estimate the relative abundance of 20 immune cell types in each sample from the GSE54238 dataset (Figure 8a). The analysis compared immune cell infiltration between HCC and normal control samples (Figure 8b). Results indicated significantly reduced proportions of macrophages M0, M1, and M2 in HCC samples, while activated memory B cells, plasma cells, CD8+ T cells, and NK cells were significantly elevated. Then, the correlation between immune cell infiltration and the expression of seven hub genes was assessed (Figure 8c). A negative correlation was observed between naïve B cells and the expression of ABCC1 and PLIN1. In contrast, macrophages M1 (*SRC*), activated mast cells, and follicular helper T cells (*AKR1B1*), as well as resting dendritic cells and regulatory T cells (*CXCL2*), showed significant positive correlations with their respective gene expression levels.

### 2.9. Molecular Docking

**Figure 9:**
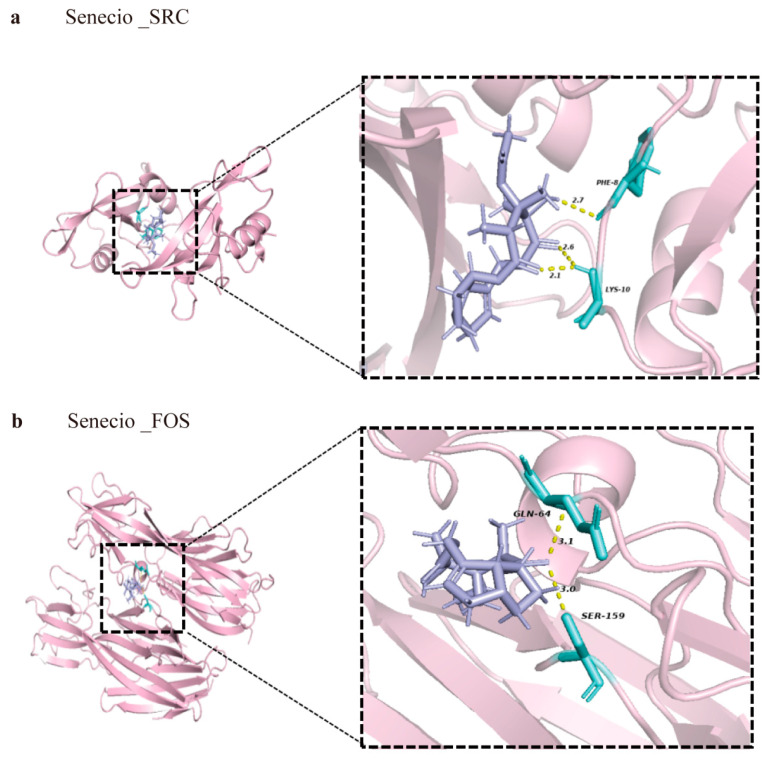
Molecular docking analysis of Climbing senecio’s active ingredients with target proteins. (**a**) Visual docking diagram of Climbing senecio–*SRC* interaction. (**b**) Visual docking diagram of Climbing senecio–*FOS*.

To further assess the potential of Climbing senecio in treating HCC, the structure of Climbing senecio was obtained from PubChem, and the docking method’s feasibility was verified through re-docking. Compound–target interactions and binding modes were visualized using PyMOL2.6.0. Key targets, *SRC* and *FOS*, were selected for molecular docking analysis. Compounds from Climbing senecio, including luteolin, quercetin, kaempferol, and artemetin, showed strong binding affinities to the target proteins. The Protein Data Bank (PDB) IDs for *SRC* and *FOS* are 1A08 and 6U3T, respectively. Binding energy calculations were used to evaluate binding affinity, where higher negative binding energy values indicate a more stable conformation and stronger spontaneous binding potential. A binding energy below −5.0 kcal/mol is generally considered indicative of a favorable binding affinity. The most favorable binding affinities were observed with *SRC* at −6.1 kcal/mol and *FOS* at −5.8 kcal/mol when they were docked with compounds from Climbing senecio (Figure 9a,b). Detailed binding analyses revealed that SRC formed three hydrogen bonds at varying distances involving two residues (PHE-8 and LYS-10). Similarly, FOS formed two hydrogen bonds at varying distances with two residues (GLN-64 and SER-159). Both targets displayed binding energies below the −5.0 kcal/mol threshold, suggesting effective binding between Climbing senecio and these receptor proteins.

## 3. Discussion

HCC is among the most common malignant tumors worldwide and remains challenging to treat effectively. Despite the potential of surgical excision, the high rates of recurrence and metastasis leading to disease-related fatalities highlight the need for improved postoperative clinical interventions. While there is extensive research on more common cancers, such as lung, breast, and colorectal cancers, fewer medicinal interventions and clinical trials focus specifically on HCC [18]. Therefore, developing novel, effective, and well-tolerated therapeutic approaches is essential to improve long-term remission following radical treatment and increase survival rates for patients with advanced HCC. Climbing senecio, a perennial herb from the Compositae family, is recognized for its therapeutic properties in TCM. It has been traditionally employed to clear heat and toxins, promote blood circulation, improve vision, reduce swelling and pain, and alleviate symptoms associated with blood stasis [19]. As such, Climbing senecio has traditionally been used to treat conditions like hepatitis, upper respiratory tract infections, cholecystitis, traumatic injuries, and inflammation from blood stasis, as well as skin sores and carbuncles. TCM has shown unique benefits in cancer prevention and treatment, particularly when used in combination with other therapeutic agents [20,21]. The bioactive compounds in plants contribute to cancer treatment by inducing programmed cell death in tumor cells through mechanisms involving caspase activation and mitochondrial dysfunction [22]. These compounds also exert anti-tumor effects by disrupting cell cycle progression at critical checkpoints, inhibiting angiogenesis, depriving tumors of essential nutrients, and preventing tumor growth and metastasis [23]. As a result, the active components of Climbing senecio may offer significant promise as part of an integrated approach to HCC therapy.

In an effort to pinpoint potential targets through which Climbing senecio may exert its effects on hepatocellular carcinoma (HCC), this investigation utilized a multi-faceted strategy that included network pharmacology, bulk RNA-seq data, and single-cell RNA-seq data. Initially, 520 potential targets of Climbing senecio were predicted and analyzed. GO analysis indicated that molecular functions related to Climbing senecio targets are primarily involved in response to xenobiotic stimulus, response to molecules of bacterial origin, and response to oxidative stress. Next, the HCC dataset from GEO was analyzed through differential expression and WGCNA to identify gene modules closely associated with clinical characteristics of HCC, resulting in 114 disease-related genes. Seven essential genes (*AKR1B1*, *CA2*, *FOS*, *CXCL2*, *SRC*, *ABCC1*, and *PLIN1*) were pinpointed at the convergence of HCC-associated genes and drug targets. A PPI network was constructed for these genes through STRING, with the MCODE algorithm employed to refine significant subsets [24]. GO analysis of these pivotal targets revealed significant enrichment in biological processes such as the apoptotic process in neurons, cellular reactions to chemical stress, and responses to oxidative stress. In terms of molecular functions, enrichment was observed in ubiquitin-like protein ligase binding, DNA-binding transcription factor binding, and RNA polymerase II-specific DNA-binding transcription factor binding. KEGG pathway analysis showed that these targets were primarily enriched in lipid and atherosclerosis, hepatitis B, and PI3K-Akt signaling pathways. Climbing senecio exerts therapeutic effects on HCC by modulating the immunological microenvironment and regulating the expression levels of the seven previously identified genes. This effect on immune cell infiltration within the tumor microenvironment highlights the potential of Climbing senecio as an effective agent in HCC treatment. GSE202642 confirmed the distribution of these important targets, with the majority (78) associated with HCC. The cell communication analysis identified complex interactions between eight cell types and HCC cells. Four signaling pathways—TGFβ (related to *SRC*), IL-1 (related to *FOS*), CXCL, and VEGF (related to *CXCL2*), were highlighted due to their significant roles in tumor occurrence, progression, and deterioration. The Src kinase, encoded by the *SRC* gene, is crucial to the TGFβ signaling pathway and promotes tumor cell invasion, metastasis, and immunosuppression, enabling tumors to evade immune surveillance. As part of the AP-1 transcription factor complex, the FOS plays a role in IL-1 signaling, which may drive HCC progression by inducing chronic inflammation, such as hepatitis. *CXCL2* attracts immune cells to the tumor site, and VEGF signaling may induce *CXCL2* secretion by TAMs and other cell types [25,26]. Angiogenesis is one of the key steps in tumor growth, and *CXCL2* may be involved in angiogenesis [27]. By cross-referencing key clusters with the intersection of Climbing senecio-ralated and HCC-related genes, we identified two primary targets: *FOS* and *SRC*. Activation of SRC, a serine/threonine kinase, can promote tumor development, cell proliferation, migration, and invasion. The *FOS* protein regulates cell growth, differentiation, and transformation, and in some contexts, *FOS* gene expression is associated with apoptotic cell death [28,29]. These two genes appear to play central roles in HCC progression and may serve as potential prognostic factors.

While this study highlights the potential impact of Climbing senecio on HCC, several limitations of the study should be acknowledged. Despite extensive pharmacological research on liver cancer, network pharmacology approaches may introduce errors when screening active compounds and action targets in TCM. Since these targets are obtained from databases, the findings rely on database accuracy and may lack support from experimental samples. Although potential targets for controlling liver cancer using Climbing senecio have been identified, further clinical pharmaceutical research is necessary to confirm the effectiveness and specificity of these targets, as various factors may influence their therapeutic effects. Bioinformatics was used in this study to analyze clinical data, with significant differences and survival analyses conducted to reduce false-positive results. Moreover, although cell communication study focuses on how signal molecules facilitate interactions between cells and their environment, cellular signaling includes various feedback loops, cross-talk, and interconnected pathways. The precise contributions of a compound-target complex are difficult to determine, as isolating its effects within these networks is challenging. Furthermore, the complexity of in vivo signaling may not be fully captured in vitro or simplified models, highlighting the need for more comprehensive animal or clinical studies. Furthermore, molecular docking was performed to evaluate and validate the binding affinity between the active constituents and their respective targets. However, this study relied on literature data and computational simulations, potentially overlooking the content variability of compounds and possible drug interactions. To confirm these findings and improve the clinical applicability of Climbing senecio, further in vitro and in vivo pharmacological experiments are necessary.

## 4. Materials and Methods

### 4.1. Identification of Climbing Senecio’s Compounds and Targets

The primary compounds in Climbing senecio were identified through searches in TCM databases: TCMBank, TCM-ID, HERB Materia Medica Identification Database, and the TCM Syndrome Association Database SymMap. Compounds related to Climbing senecio were uploaded to TCMSP for retrieval. For screening, compounds with oral bioavailability (OB) ≥ 30% and drug-likeness (DL) ≥ 0.18 were selected. Known and potential targets of Climbing senecio were identified through the SwissTargetPrediction online database and the TCMSP platform. Additional potential targets were predicted using the BindingDB and TargetNet databases. The functional enrichment analyses for these Climbing senecio targets were implemented utilizing the “clusterProfiler” R package 4.3.1 [30]. The biological properties of these targets were explored through 3 GO analysis categories. Potential signaling pathways were identified using KEGG enrichment analysis.

### 4.2. Identification of DEGs in HCC

The mechanism by which persistent liver inflammation may promote the development of cancer through the expansion of tumor-initiating cells is not clear. GSE54238 is a dataset measuring the expression profile of lncRNA/mRNA in normal, chronic hepatitis, liver cirrhosis, and cancerous liver. The platforms for GSE54238 are derived from GPL16955 (Arraystar human lncRNA microarray V1-100309 [gene-level version]), with the technology type being in situ oligonucleotide, and the organism studied is *Homo sapiens*. This dataset is of the type Expression profiling by array and Non-coding RNA profiling by array. The GSE54238 microarray dataset from the GEO database (http://www.ncbi.nlm.nih.gov/geo, accessed on 18 March 2024) was generated by Shengxian Yuan et al., from an HCC cohort comprising 10 healthy samples and 46 disease samples collected from two regions, China and South Korea. The dataset included 56 male samples with an age range of 26–65 years (Supplementary Table S4) [31]. Raw data were first standardized. DEGs between disease samples and healthy controls were discovered using the “limma” package in R, with criteria of |fold change| (FC) > 1 and *p* < 0.05. A heatmap was generated to highlight key genes and visualize DEGs [32]. Gene set enrichment analysis (GSEA) was used to identify specific genomic features and to explore distinctions between biological processes in DEGs.

### 4.3. Weighted Gene Co-Expression Network Analysis

The construction of a gene co-expression network within the GSE54238 dataset was executed utilizing the “WGCNA” package in the R programming environment [33]. Hierarchical clustering trees were generated to handle outlier samples. The correlation matrix and topological overlap between genes were then calculated. The soft-threshold power was ascertained by employing the “pickSoftThreshold” function [34]. To ensure that the network follows a scale-free topology, the paired Pearson correlation matrix of each gene was converted into a neighborhood correlation matrix according to the predetermined screening threshold. The eigenvector values of each module were calculated, and the adjacency matrix was converted into a topological overlap matrix (TOM). The corresponding dissimilarity was then computed, followed by hierarchical clustering analysis. Essential modules were identified by assessing the association between gene modules and sample types (normal and tumor). We analyzed the correlation between the characteristic genes of different modules, as well as their correlation with the sample phenotype, in order to identify the modules related to the pathological process of HCC.

### 4.4. Construction of Protein–Protein Interaction Networks and Recognization of Key Clusters

PPIs of pharmacological targets and HCC-related genes were analyzed using the STRING database (https://string-db.org/, accessed on 3 April 2024), with PPI evaluation based on network density and average degree [35]. Interactions between proteins were visualized through the network nodes and edges of the PPI network. The MCODE algorithm was applied to identify primary targets involved in HCC growth and proliferation, with Cytoscape3.10.1 software used to further enhance and analyze the PPI networks [36]. The cutoff degree for the network scoring module of the MCODE algorithm was set to 2, with a node score cutoff of 0.2 for the clustering finding module, k-score = 2, and Max. Depth = 100.

### 4.5. Transcriptome Difference Evaluation and ROC Curve Evaluation of Key Clusters

The key targets were re-evaluated within the GSE54238 dataset to assess expression differences between HCC and control groups. The criteria for screening were established with a significance level of *p* < 0.05 and a fold-change magnitude exceeding 1 as indicated by |log2(fold-change)| > 1. Kaplan–Meier (K-M) analysis, ROC curve generation, and AUC calculation were conducted using the “pROC” and “ggplot2” packages in R [37]. These evaluations were designed to assess the prognostic relevance of core target genes regarding patient outcomes. AUC measurement, ROC-based diagnostic accuracy, and survival analysis provided a comprehensive evaluation of these biomarkers’ predictive potential in HCC [38]. For survival analysis, patient survival statistics from The Cancer Genome Atlas(TCGA) database (http://xena.ucsc.edu/, accessed on 25 March 2024) were also incorporated.

### 4.6. Analysis of Single-Cell RNA Sequencing Data and Identification of Genes Associated with HCC

Raw data from GSE202642 were downloaded from the GEO database for single-cell RNA sequencing analysis and identification of HCC-related genes. GSE202642 employs the scRNA-seq method to profile a large number of cells from primary tumors. The dataset includes 74,957 single-cell sequencing entries from 7 tumors of untreated patients with hepatitis B (HBV)-related HCC, as well as adjacent liver tissues from 4 patients [39]. Seven HCC tissue samples were analyzed with strict quality control measures, including data standardization, cell cycle correction, batch effect elimination, and removal of mitochondrial genes and outliers. The “Seurat” package was used to standardize data [40,41]. Parameters set for cell selection included min.cells = 5, min.features = 200, af$Feature_RNA ≥ 300 and af$Feature_RNA ≤ 7000, af$percent.mt ≤ 20%, and af$percent.rb ≤ 20%. After filtering, selected cells were grouped and categorized for graphical representation. The “singscore” package in R was utilized to graphically represent the distribution of pharmacologically pertinent targets across various cell subtypes.

### 4.7. Cell Communication Analysis

Intercellular communication analysis was performed using annotated data to investigate interactions between different cell types [42]. The “CellChat” package in R was used for intercellular communication analysis, with the “patchwork” package employed for visualizing related charts. We designated the “CellChatDB.human” as our ligand–receptor interaction repository. Subsequently, the “computeCommunication” function was employed to determine the likelihood of cell-to-cell interactions. Furthermore, the “filterCommunication” function was applied to filter out any interactions that were not observed in at least 10 cells. The “computeCommunProbPathway” function was used to evaluate cell-to-cell communication probability at the signaling pathway level, and the “subsetCommunication” function was used to construct the CellChat network.

### 4.8. Immune Infiltration Analysis

The CIBERSORT algorithm (https://cibersortx.stanford.edu/, accessed on 6 April 2024) was employed to assess the potential correlation between target hub genes and alterations in the immune microenvironment of HCC patients [43]. Immunological scores for each sample were calculated using the ESTIMATE method, and the relative proportions of 20 immune cell types were estimated based on the GSE54238 dataset. Furthermore, Spearman correlation analysis was performed to investigate the relationship between the hub genes and immune cell types.

### 4.9. Molecular Docking

The crystal structures of *FOS* (PDB ID: 6U3T) and *SRC* (PDB ID: 1A08) were downloaded from the PDB for molecular docking. Protein structures with bound active site inhibitors were preferred to improve simulation accuracy. The structure of Climbing senecio was obtained from PubChem (PubChem CID: 259727). Firstly, an open-source software, PyMOL 2.6.0, was used to remove ligand residues and water molecules from the protein structures to ensure structural purity. Then, hydrogen atoms were added to the protein structures employing AutoDock Tools 1.5.6., and charges were calculated. The processed macromolecular proteins were saved in pdbqt format for use as receptors in molecular docking [44]. The prepared pdbqt files for receptor and ligand were then imported into AutoDock Tools 1.5.6. Gridbox parameters, including three-dimensional center coordinates (x, y, z) and dimensions, were set for the docking box. The autogrid and autodock script files were run for molecular docking, employing the Local Search Parameters algorithm to generate the docking parameter file (dpf). The molecular docking results were visualized using PyMOL 2.6.0 Binding energy values were used to evaluate the docking results, with binding energies less than 0 kcal/mol indicating binding activity between the protein and ligands and values less than −5.0 kcal/mol suggesting strong binding activity [45].

## 5. Conclusions

Intending to provide new ideas for the regulation and control of HCC, this study searched for the main active components and possible targets of Climbing senecio scandens by means of bioinformatics. The Climbing senecio–HCC interaction network and functional enrichment analyses suggest that multi-targeted HCC treatment with Climbing senecio involves numerous biological processes. Cross-analysis identified *AKR1B1*, *CA2*, *FOS*, *CXCL2*, *SRC*, *ABCC1*, and *PLIN1* as overlapping targets between Climbing senecio and HCC. Using the MCODE algorithm, a core cluster was identified, with *SRC* and *FOS* emerging as central target genes. Single-cell sequencing analysis revealed that Climbing senecio’s targets primarily influenced HCC clusters, stromal cells, and dendritic cells. Cell communication analysis highlighted four key pathways involving *SRC*, *FOS*, and *CXCL2* that influence HCC: TGFβ, IL-1, VEGF, and CXCL signaling. Immune infiltration analysis of HCC indicates the presence of a unique immune microenvironment within the tumor compared to normal liver tissue. Molecular docking results demonstrated that the two hub target proteins, *SRC* and *FOS*, show favorable binding affinity with Climbing senecio. These findings illustrate the molecular mechanisms and pathways by which Climbing senecio may exert anti-HCC effects and offer new insights into multi-targeted drug strategies for HCC treatment. However, acting on the same targets in tumor cells, or on different cells in the microenvironment, may have different effects on tumor progression; further validation through cell, animal, and clinical studies is required to confirm these results.

## Data Availability

The datasets (GSE54238 and GSE202642) analyzed in this study are available from the Gene Expression Omnibus database repository (https://www.ncbi.nlm.nih.gov/geo/, accessed on 18 March 2024). Other relevant raw data can be provided upon reasonable request (e.g., for further analysis).

## Supplementary Materials

The following supporting information can be downloaded at: https://www.mdpi.com/article/10.3390/ph17121707/s1, Table S1: Candidate Active ingredients of *Senecio scandens* Buch.-Ham; Table S2: Four databases of targets related to *Senecio scandens* Buch.-Ham; Table S3: The differential expression genes and fold change and genes associated with HCC; Table S4: GSE54238 Clinical Processing Data Table; Figure S1: Filter cell standards; Figure S2: Survival analysis.

## Abbreviations

Climbing senecio	*Senecio scandens* Buch.-Ham
HCC	hepatocellular carcinoma
GEO	Gene Expression Omnibus
HBV	hepatitis B
HBC	hepatitis C
TCMSP	Traditional Chinese Medicine Systems Pharmacology Database and Analysis Platform
DEGs	differentially expressed genes
WGCNA	weighted gene correlation network analysis
PPI	protein–protein interaction
STRING	Search Tool for the Retrieval of Interacting Genes/Protein
GO	Gene Ontology
BP	biological process
CC	cellular component
MF	molecular function
KEGG	Kyoto Encyclopedia of Genes and Genomes
PDB	Protein Data Bank
ROC	receiver operating characteristic curve
TCM	traditional Chinese medicine
MCODE	molecular complex detection
TCGA	The Cancer Genome Atlas
