# Supplementary material for: Single-Cell RNA Sequencing, Cell Communication, and Network Pharmacology Reveal the Potential Mechanism of Senecio scandens Buch.-Ham in Hepatocellular Carcinoma Inhibition
Source: Pharmaceuticals (Basel). 2024 Dec 18;17(12):1707. doi: 10.3390/ph17121707 (PMC11676315; doi:10.3390/ph17121707)

Supplementary Table S1. Candidate Active ingredients of *Senecio scandens* Buch.-Ham

MOL ID	Molecule Name	IUPAC chemical name	OB%	DL	Chemical Structure Depiction
MOL010023	senkirkine	(1R,4Z,6R,7R,11Z)-4-ethylidene-7-hydroxy-6,7,14-trimethyl-2,9-dioxa-14-azabicyclo[9.5.1]heptadec-11-ene-3,8,17-trione	56.16	0.41	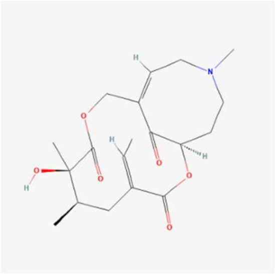
MOL002680	Flavoxanthin	(2R,6S,7aR)-2-[(2E,4E,6E,8E,10E,12E,14E,16E)-17-[(1R,4R)-4-hydroxy-2,6,6-trimethylcyclohex-2-en-1-yl]-6,11,15-trimethylheptadeca-2,4,6,8,10,12,14,16-octaen-2-yl]-4,4,7a-trimethyl-2,5,6,7-tetrahydro-1-benzofuran-6-ol	60.41	0.56	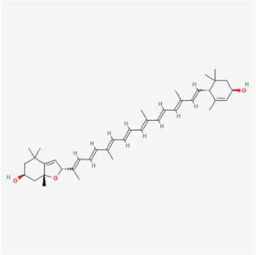
MOL004492	Chrysanthemaxanthin	(2S,6S,7aR)-2-[(2E,4E,6E,8E,10E,12E,14E,16E)-17-[(1R,4R)-4-hydroxy-2,6,6-trimethylcyclohex-2-en-1-yl]-6,11,15-trimethylheptadeca-2,4,6,8,10,12,14,16-octaen-2-yl]-4,4,7a-trimethyl-2,5,6,7-tetrahydro-1-benzofuran-6-ol	38.72	0.58	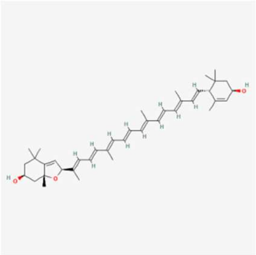

MOL000006	Luteolin	2-(3,4-dihydroxyphenyl)-5,7-dihydroxychromen-4-one	36.16	0.25	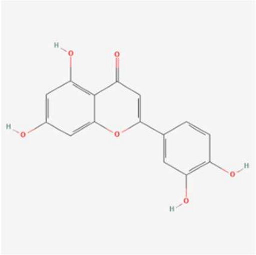
MOL000665	Flemiphilippinin C	5-hydroxy-7-(4-hydroxy-3-methoxyphenyl)-2,2-dimethyl-10-(3-methylbut-2-enyl)pyrano[3,2-g]chromen-6-one	47.66	0.73	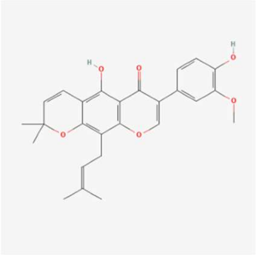
MOL000098	quercetin	2-(3,4-dihydroxyphenyl)-3,5,7-trihydroxychromen-4-one	46.43	0.28	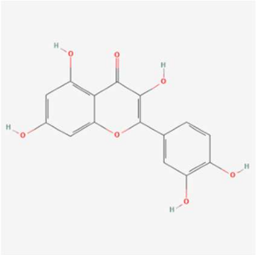
MOL000422	kaemferol	3,5,7-trihydroxy-2-(4-hydroxyphenyl)chromen-4-one	41.88	0.24	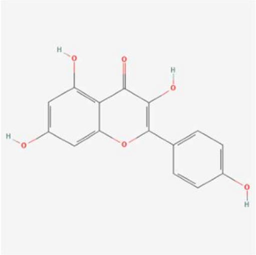
MOL000211	Betulinic Acid	(1R,3aS,5aR,5bR,7aR,9S,11aR,11bR,13aR,13bR)-9-hydroxy-5a,5b,8,8,11a-pentamethyl-1-prop-1-en-2-yl-1,2,3,4,5,6,7,7a,9,10,11,11b,12,13,13a,13b-hexadecahydrocyclopenta[a]chrysene-3a-carboxylic acid	55.38	0.78	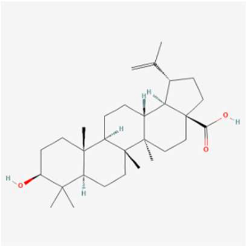

MOL004798	Anthocyanin	2-(3,4-dihydroxyphenyl)chromenylium-3,5,7-triol	40.63	0.28	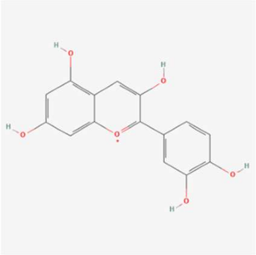
MOL001790	Linarin	5-hydroxy-2-(4-methoxyphenyl)-7-[(2S,3R,4S,5S,6R)-3,4,5-trihydroxy-6-[[(2R,3R,4R,5R,6S)-3,4,5-trihydroxy-6-methyloxan-2-yl]oxymethyl]oxan-2-yl]oxychromen-4-one	39.84	0.71	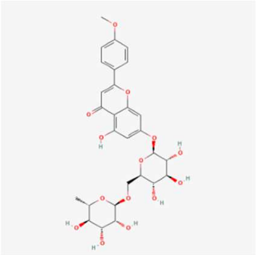
MOL005229	Artemetin	2-(3,4-dimethoxyphenyl)-5-hydroxy-3,6,7-trimethoxychromen-4-one	49.55	0.48	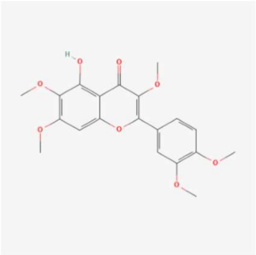

Supplementary Table S2. Four databases of targets related to *Senecio scandens* Buch.-Ham

Binding DB	Swiss Target Prediction	TargetNet	TCMSP	DRUG
HSD17B1	JAK3	CYP11B2	PTGS2	HSD17B1
ACHE	JAK1	CES1	NCOA2	ACHE
AKR1B1	JAK2	AKR1B1	PTGS1	AKR1B1
SNCA	CFD	ACHE	AR	SNCA
MAOB	PSEN2	MAOB	HSP90AA1	MAOB
AR	JAK3	PGR	PRSS1	AR
CYP19A1	ADORA2A	MAOA	DPP4	CYP19A1
ABCB1	ADORA3	S1PR4	RELA	ABCB1
AURKB	MAP2K1	CYP2C9	EGFR	AURKB
BACE1	CXCR2	JAK2	AKT1	BACE1
ABCG2	IDO1	L3MBTL1	VEGFC	ABCG2
BRD4	PDE10A	MAP3K10	CCND1	BRD4
CA12	ABCC9	HTR2C	BCL2L1	CA12
CA2	KIF11	APOBEC3A	CDKN1A	CA2
CA4	HCRTR2	ADRA1D	CASP9	CA4

CA7	HTR1D	PIM1	MMP2	CA7
CBR1	PIK3CA	DRD5	MMP9	CBR1
CSNK2A1	GSK3B	HCRTR1	MAPK1	CSNK2A1
CSNK2A3	ADAM17	DRD1	IL10	CSNK2A3
CCNC	CASP8	CES2	RB1	CCNC
CDK1	CASP1	HSP90AB1	CDK4	CDK1
CYP1A1	LRRK2	CDK7	TNF	CYP1A1
CYP1A2	CDK5R1	EGFR	JUN	CYP1A2
TOP1	CDK2	APP	IL6	TOP1
TOP2A	DYRK1A	KDR	CASP3	TOP2A
APLF	HSD17B2	SRC	TP53	APLF
ELAVL3	HCRTR1	HTR1A	NFKBIA	ELAVL3
EGFR	DGAT1	LCK	XDH	EGFR
ESR2	MAPK14	HSD17B3	TOP1	ESR2
GSK3B	PDE4B	THRB	MDM2	GSK3B
MET	PDE7A	AR	APP	MET
IP6K2	PPP1CA	LTB4R	MMP1	IP6K2
EMD	HDAC6	CNR2	PCNA	EMD
GLO1	HDAC1	CHRM5	ERBB2	GLO1
MMP12	BDKRB1	CA14	PPARG	MMP12
MMP9	ADORA2B	CYP3A4	HMOX1	MMP9
MBNL1	NTRK1	CACNA1B	CASP7	MBNL1
NOX4	SYK	HTR7	ICAM1	NOX4
PSIP1	FARS2	ROCK2	MCL1	PSIP1
PIK3CG	TK1	FYN	BIRC5	PIK3CG
TNKS2	NPY5R	HSD17B2	IL2	TNKS2
F2	FAP	ABCB1	CCNB1	F2
PKM	PDE5A	PTGS2	TYR	PKM
FLT3	FYN	PTK2	IFNG	FLT3
PIM1	YES1	PTPN22	IL4	PIM1
SLCO2B1	AURKB	CYP1A2	TOP2A	SLCO2B1
SI	PYGL	RAC1	GSTP1	SI
SULT1A1	SRC	TUBB2B	XIAP	SULT1A1
YES1	AURKA	ALOX5	SLC2A4	YES1
KDR	HPGDS	DUSP3	INSR	KDR
XDH	DRD4	RELA	CD40LG	XDH
NEU4	CTSD	CYP19A1	PTGES	NEU4
HSD17B2	PDE2A	XDH	NUF2	HSD17B2
ADORA1	KDR	NOS2	ADCY2	ADORA1
ADORA3	BACE1	PTGS1	MET	ADORA3
AHR	EIF4A1	MIF	SERPIND1	AHR
CISD1	ROCK2	ALOX15	ESR1	CISD1
TRPC5	F8	AHR	F7	TRPC5
TST	GRM5	NR2E3	KDR	TST

ALB	ROCK1	BCL2A1	ACHE	ALB
NR1H4	MET	ABCG2	TOP2B	NR1H4
GPBAR1	MAPK1	RORA	ESR2	GPBAR1
CES1	SCN9A	CA7	GSK3B	CES1
F3	MYLK	TUBA1A	CCNA2	F3
IL2	PDE9A	RPS6KA3	CALM3	IL2
TNF	P2RX7	CA6	KCNH2	TNF
	KDM1A	ALPL	PLAU	PTGS2
	CCND1	TLR9	PON1	NCOA2
	CYP19A1	PDE7A	CTSD	PTGS1
	CCNE2	PTPN7	SULT1E1	HSP90AA1
	AMPD3	GPR35	EGF	PRSS1
	PER2	FLT3	CCL2	DPP4
	IRAK4	PDE4D	IL1B	RELA
	CNR2	NR2F2	SELE	AKT1
	PARP1	FLT4	MPO	VEGFC
	PARP3	PLIN5	CDK1	CCND1
	CDK1	TYK2	PLAT	BCL2L1
	HSP90AB1	TERT	GJA1	CDKN1A
	MDM2	HTR1E	HSPA5	CASP9
	CASP3	MCL1	ACPP	MMP2
	SIGMAR1	ESR2	VCAM1	MAPK1
	CASP7	CA4	MMP3	IL10
	TYK2	CA13	THBD	RB1
	LIPG	PDE4A	F3	CDK4
	KCNE1	CLK1	NQO1	JUN
	RPS6KA2	DYRK1A	GSTM2	IL6
	BRPF1	HSD17B1	SCN5A	CASP3
	BRD4	PDE3A	BCL2	TP53
	MMP3	PLIN1	ALOX5	NFKBIA
	STAT3	GSK3B	COL1A1	MDM2
	CREBBP	HNF4A	NOS3	APP
	EIF2AK3	CYP17A1	COL3A1	MMP1
	PTK2	CTDSP1	MAOB	PCNA
	CYP17A1	PDGFRA	POR	ERBB2
	CAPN2	DRD2	PTGER3	PPARG
	CAPN1	CHRM4	SOD1	HMOX1
	OPRL1	ADRA2C	CYP1A2	CASP7
	NCOR2	PLA2G1B	ODC1	ICAM1
	REN	PDE4B	CYP3A4	MCL1
	NOX4	IMPDH2	RXRA	BIRC5
	AKR1B1	NR3C2	AHR	CCNB1
	XDH	TNF	ACACA	TYR
	MAOA	PTPN1	ADRB2	IFNG

FLT3	TEK	AKR1B1	IL4
CA2	CA12	GABRA1	GSTP1
CCNB3	PLK1	GSTM1	XIAP
ALOX5	ESR1	MGAM	SLC2A4
ADORA1	DNMT1	FOS	INSR
CA7	S1PR2	EIF6	CD40LG
GLO1	HTR1B	BAX	PTGES
APP	S1PR1	AHSA1	NUF2
TTR	KCNC3	ELK1	ADCY2
MMP9	CASP9	CASP8	SERPIND1
CA12	THRA	PRKCA	ESR1
MMP2	APOBEC3G	HIF1A	F7
CA4	PTPRC	STAT1	TOP2B
MMP12	CA5A	RUNX1T1	CCNA2
CD38	CYP2C19	CAV1	CALM3
CYP1B1	HTR6	MYC	KCNH2
ABCG2	GALR3	CYP1A1	PLAU
AKR1B10	CA5B	CXCL8	PON1
TNKS2	SLC5A2	PRKCB	CTSD
TNKS	SLC5A1	DUOX2	SULT1E1
TOP1	CA9	HSPB1	EGF
ARG1	MMP12	TGFB1	CCL2
PTPRS	HSP90AA1	NR1I2	IL1B
ABCC1	ADORA1	CYP1B1	SELE
HSD17B1	SERPINE1	SERPINE1	MPO
ACHE	CDC42	IL1A	PLAT
CDK6	STS	NCF1	GJA1
ABCB1	EDNRA	ABCG2	HSPA5
ESR2	ADORA2A	HAS2	ACPP
CSNK2A1	TOP1	NFE2L2	VCAM1
ALOX15	ADK	PARP1	MMP3
ALOX12	SLC6A3	PSMD3	THBD
ESR1	MGLL	CXCL11	NQO1
PTGS2	SLC6A2	CXCL2	GSTM2
CFTR	SLC6A4	DCAF5	SCN5A
AMY1A	HTR5A	NR1I3	BCL2
GRK6	CHRNA7	CHEK2	ALOX5
CA1	CHRNA4	CLDN4	COL1A1
CA9	PLAT	PPARA	NOS3
TERT	DHODH	PPARD	COL3A1
TYR	TAAR1	HSF1	POR
AHR	NOS1	CRP	PTGER3
ESRRA	PTGES	CXCL10	SOD1
GPR35	GRIN2B	CHUK	ODC1

AVPR2	GSK3A	SPP1	CYP3A4
IGF1R	GRM5	RUNX2	RXRA
EGFR	PLAU	RASSF1	ACACA
F2	FAAH	E2F1	ADRB2
PIM1	NOS3	E2F2	GABRA1
MPO	SIGMAR1	IGFBP3	GSTM1
PIK3R1	ROCK1	IGF2	MGAM
DAPK1	GRM4	IRF1	FOS
MMP13	CDC7	ERBB3	EIF6
CA3	PDGFRB	DIO1	BAX
PLK1	ADRA2B	PCOLCE	AHSA1
CA6	HCAR2	NPEPPS	ELK1
PKN1	FDPS	HK2	CASP8
CA14	CYP11B1	NKX3-1	PRKCA
NEK2	HDAC4	RASA1	HIF1A
CXCR1	HRH1	NOS2	STAT1
CAMK2B	ADRA2A	PGR	RUNX1T1
ALK	F10	CHRM1	CAV1
AKT1	HTR3A	SLC6A2	MYC
NEK6	ATP4A	CHRM2	CXCL8
PLA2G1B	KIF11	ADRA1B	PRKCB
CA5A	CDC25B	IKBKB	DUOX2
AXL	CTSG	MAPK8	HSPB1
NUAK1	CHEK2	AKR1C3	TGFB1
AKR1C2	CSNK2A1	SLPI	NR1I2
AKR1C1	GRIN1	CA2	CYP1B1
AKR1C3	HTR2A	MAPK14	SERPINE1
AKR1C4	CHRM3	CDK2	IL1A
CA13	SIRT2	CHEK1	NCF1
AKR1A1	PRSS1		HAS2
PFKFB3	NR1H3		NFE2L2
PLG	TSPO		PARP1
KDM4E	PKIA		PSMD3
AR	SIRT1		CXCL11
PTPN1	CHRFAM7A		CXCL2
RARA	CYP2D6		DCAF5
F10	GRIA2		NR1I3
MAPT	MAPK14		CHEK2
TOP2A	PDE10A		CLDN4
INSR	HSD11B2		PPARA
PIK3CG	PTPN2		PPARD
APEX1	NR1H4		HSF1
MPG	GPBAR1		CRP
SLC22A12	SRD5A2		CXCL10

CDK5R1	CHRM1	CHUK
CCNB3	ACE	SPP1
TNF	HSD11B1	RUNX2
IL2	CHRM2	RASSF1
NMUR2	S1PR5	E2F1
ADRA2A	SRD5A1	E2F2
ADRA2C	FDFT1	IGFBP3
ALDH2	PREP	IGF2
RPS6KA3	AVPR1A	IRF1
NQO2	RXRB	ERBB3
TP53	RARA	DIO1
OPRD1	OPRM1	PCOLCE
MCL1	PRKCE	NPEPPS
OPRM1	CA1	HK2
ODC1	PRKCG	NKX3-1
PLA2G2A	HDAC2	RASA1
KIT	TRPV1	NOS2
ST6GAL1	FOLH1	PGR
NOS2	CDK1	CHRM1
BCHE	OPRD1	SLC6A2
POLB	CDK2	CHRM2
SAE1	PRKCB	ADRA1B
HSD11B1	CDK5	IKBKB
PTGES	CA2	MAPK8
NR1H4	CXCR2	AKR1C3
PTPN2	RARG	SLPI
CDC25C	MDM2	MAPK14
GPBAR1	MMP9	CDK2
RORC	PIK3CG	CHEK1
PTPRF	BCHE	JAK3
ACP1	CACNA1H	JAK1
CDC25B	PIM3	JAK2
CES2	EPHB4	CFD
CDC25A	MAPKAPK2	PSEN2
PDE4D	RARB	ADORA2A
FABP1	RXRG	MAP2K1
UGT2B7	RXRA	CXCR2
VDR	KCNH2	IDO1
SCD	LACTBL1	PDE10A
SERPINA6	NR3C1	ABCC9
SHBG	METAP2	KIF11
G6PD	NTRK1	HCRTR2
CYP51A1	HTR1F	HTR1D
HSD17B3	HTR4	PIK3CA

GABBR1	HRH2	ADAM17
PPARG		CASP1
PRKCH		LRRK2
HSD11B2		CDK5R1
PTPN11		DYRK1A
NPC1L1		HCRTR1
ALOX5AP		DGAT1
GABRA2		PDE4B
PTPN6		PDE7A
TLR9		PPP1CA
PTGER2		HDAC6
FABP2		HDAC1
THRA		BDKRB1
THRB		ADORA2B
		NTRK1
		SYK
		FARS2
		TK1
		NPY5R
		FAP
		PDE5A
		FYN
		PYGL
		SRC
		AURKA
		HPGDS
		DRD4
		PDE2A
		EIF4A1
		ROCK2
		F8
		GRM5
		ROCK1
		SCN9A
		MYLK
		PDE9A
		P2RX7
		KDM1A
		CCNE2
		AMPD3
		PER2
		IRAK4
		CNR2
		PARP3

HSP90AB1
SIGMAR1
TYK2
LIPG
KCNE1
RPS6KA2
BRPF1
STAT3
CREBBP
EIF2AK3
PTK2
CYP17A1
CAPN2
CAPN1
OPRL1
NCOR2
REN
MAOA
CCNB3
TTR
CD38
AKR1B10
TNKS
ARG1
PTPRS
ABCC1
CDK6
ALOX15
ALOX12
CFTR
AMY1A
GRK6
CA1
CA9
TERT
ESRRA
GPR35
AVPR2
IGF1R
PIK3R1
DAPK1
MMP13
CA3
PLK1

CA6
PKN1
CA14
NEK2
CXCR1
CAMK2B
ALK
NEK6
PLA2G1B
CA5A
AXL
NUAK1
AKR1C2
AKR1C1
AKR1C4
CA13
AKR1A1
PFKFB3
PLG
KDM4E
PTPN1
RARA
F10
MAPT
APEX1
MPG
SLC22A12
NMUR2
ADRA2A
ADRA2C
ALDH2
RPS6KA3
NQO2
OPRD1
OPRM1
PLA2G2A
KIT
ST6GAL1
BCHE
POLB
SAE1
HSD11B1
PTPN2
CDC25C

RORC
PTPRF
ACP1
CDC25B
CES2
CDC25A
PDE4D
FABP1
UGT2B7
VDR
SCD
SERPINA6
SHBG
G6PD
CYP51A1
HSD17B3
GABBR1
PRKCH
HSD11B2
PTPN11
NPC1L1
ALOX5AP
GABRA2
PTPN6
TLR9
PTGER2
FABP2
THRA
THRB
CYP11B2
S1PR4
CYP2C9
L3MBTL1
MAP3K10
HTR2C
APOBEC3A
ADRA1D
DRD5
DRD1
CDK7
HTR1A
LCK
LTB4R
CHRM5

CACNA1B
HTR7
PTPN22
RAC1
TUBB2B
DUSP3
MIF
NR2E3
BCL2A1
RORA
TUBA1A
ALPL
PTPN7
NR2F2
FLT4
PLIN5
HTR1E
PDE4A
CLK1
PDE3A
PLIN1
HNF4A
CTDSP1
PDGFRA
DRD2
CHRM4
IMPDH2
NR3C2
TEK
DNMT1
S1PR2
HTR1B
S1PR1
KCNC3
APOBEC3G
PTPRC
CYP2C19
HTR6
GALR3
CA5B
SLC5A2
SLC5A1
CDC42
STS

EDNRA
ADK
SLC6A3
MGLL
SLC6A4
HTR5A
CHRNA7
CHRNA4
DHODH
TAAR1
NOS1
GRIN2B
GSK3A
FAAH
GRM4
CDC7
PDGFRB
ADRA2B
HCAR2
FDPS
CYP11B1
HDAC4
HRH1
HTR3A
ATP4A
CTSG
GRIN1
HTR2A
CHRM3
SIRT2
NR1H3
TSPO
PKIA
SIRT1
CHRFAM7A
CYP2D6
GRIA2
SRD5A2
ACE
S1PR5
SRD5A1
FDFT1
PREP
AVPR1A

				RXRB
				PRKCE
				PRKCG
				HDAC2
				TRPV1
				FOLH1
				CDK5
				RARG
				CACNA1H
				PIM3
				EPHB4
				MAPKAPK2
				RARB
				RXRG
				LACTBL1
				NR3C1
				METAP2
				HTR1F
				HTR4
				HRH2

Supplementary Table S3. The differential expression genes and fold change and genes associated with HCC

Decreased expression genes	logFC	Increased expression genes	logFC	114 genes associated with HCC
AK055863	−2.358001361	STX6	1.373243758	AAK1
AK021739	−1.291925073	GYG1	1.420736826	ABCC1
CNDP1	−3.11171939	AAK1	1.11623125	ABCC5
VIPR1	−2.282908662	VGLL4	1.151620332	ADRA1A
SULT1E1	−2.163501845	EXOC2	1.178751185	AK021739
USH2A	−2.611634132	SLC36A1	1.303780967	AK055863
AK094196	−1.772540948	TESK1	1.390502788	AK094127
BC005927	−2.375042892	C1orf216	1.065161364	AK094196
AADAT	−2.235576249	LOC541471	1.25685054	AK123630
TUBE1	−1.346513521	ZCCHC17	1.129485659	AKR1B1
DBH	−2.444314926	UTP18	1.290855216	AKR1CL1
PLIN1	−2.27459752	RNF219	1.12219884	ANGPTL6
ST3GAL6	−1.50878348	GAL3ST4	1.325629883	ASPA
SLC9B2	−1.971607131	FAM116A	1.147330909	ASPG
HAMP	−2.69206085	TMEM51	1.277866083	AURKAPS1

RAPH1	-1.158194317	ACVRL1	1.063202959	BC014180
ARHGAP10	-1.137052957	AURKAPS1	1.521295002	BC030271
ADRA1A	-2.155813215	E2F3	1.018441772	BC035094
ADAMTS13	-1.729983578	PPP1R2	1.175568363	BMP5
CLEC1B	-2.463306438	LOC151162	1.240449871	BRP44L
CACNA1H	-1.978970515	MFAP1	1.095350171	C14orf129
AF143870	-1.685349401	MRPS23	1.319123565	C1orf216
LCAT	-1.882070502	SLAMF8	1.178794893	CA2
SLC3A1	-1.390342809	PHLDA3	1.040570957	CHMP4C
CYP2B7P1	-2.112483028	DCAF16	1.24143667	CLEC1B
ABCC9	-2.151918532	KIAA1702	1.055536974	CLEC4G
AK126253	-2.169940802	C9orf167	1.176410037	CLIC1
DAB1	-1.525284622	PDGFA	1.839628514	CR602022
SLC45A3	-1.096579347	H2AFZ	1.058177559	CR613718
HSD17B13	-3.41615323	TAP1	1.250364394	CRHBP
PTH1R	-2.096777397	GSTA4	1.09876052	CSAD
SLC6A13	-1.989834239	LPCAT1	1.546006441	CXCL2
BBOX1	-2.237238904	RHEB	1.116754541	DBH
AQP7P1	-1.32749747	AK094127	1.080765925	DCAF16
FCN3	-2.601489303	ITGAV	1.038347345	DIO3AS
AK124928	-2.026582891	TTYH3	1.030381991	DUSP1
FOLH1B	-1.905117951	CR602022	1.073134859	E2F3
LOC90586	-1.784908394	TMEM184B	1.28971073	ECM1
ADK	-1.607327995	BLVRA	1.448903207	EDARADD
MOGAT1	-1.979938909	GPNMB	1.0227817	EGR1
TPPP2	-1.709859284	MANF	1.154860484	EXOC2
DKFZp781M09150	-2.489487524	PIR	1.117064124	FAM116A
ABCA9	-1.298908802	HSPA4L	1.171585182	FAM180A
BC014180	-1.110363833	LAPTM4B	1.579625054	FBL3B
CLEC4G	-2.295309127	EDARADD	1.092454307	FCN2
IQGAP2	-1.140858909	PRDM1	1.085015963	FCN3
CYP4F3	-2.528499687	CDC7	1.234131422	FOS
ACSM3	-1.947127029	YEATS2	1.119219769	FOSB
THRSP	-3.006389107	SIGLEC8	1.058704106	GAL3ST4
CSAD	-1.604672703	METTL1	1.048618869	GCH1
GBA3	-2.43913893	TFRC	1.2257633	GDF2
FOLH1	-2.036802311	CLIC1	1.001182326	GDPD4
C14orf167	-1.092617972	GPC3	3.417451585	GLUD2
FAM134B	-1.603676237	BC030271	1.220246203	GPC3
ID1	-1.26425857	SORT1	1.461404954	GPNMB
ALB	-2.069968405	LY96	1.268724829	GSTA4
FCN2	-2.341415265	WFS1	1.127984554	GYG1
RCL1	-1.78903057	MCAM	1.377793493	H2AFZ
GLUD2	-1.573258787	TPRKB	1.024564077	HAMP

GDPD4	-1.234509249	KPNA2	1.797059166	HAND2
GNE	-1.596646433	ANXA2P3	1.268866657	HSPA4L
GHR	-2.08664095	AKR1B1	1.061918253	IGFALS
GNAO1	-1.287966715	TRAF5	1.182722628	IL27
RGPD3	-1.115552175	ABCC5	1.166498125	KIAA1702
KMO	-1.59556267	RAI1	1.00200563	KIF21B
DAK	-1.637318139	ABCC1	1.090148634	KMO
RGPD1	-1.813882551	KIF21B	1.37426741	KPNA2
AKR1CL1	-1.839270111	LMCD1	1.481682423	LAPTM4B
LOC255167	-2.634967005	UBD	1.732245148	LCAT
ZBTB16	-1.260216542	RIMBP3C	1.017294877	LOC151162
GCH1	-1.281751391	CHMP4C	1.073279795	LOC541471
RGPD6	-1.435015326	C14orf129	1.079447868	LOC90586
PLGLB2	-1.993117353	STK39	1.390740552	MANF
ASPA	-1.518333687	SLC2A6	1.004367023	MARCO
F11	-2.440448739	NPNT	1.33375533	MBL1P
EGR1	-1.872143082	SRC	1.223612086	METTL1
ECHDC2	-1.444097737	ZSWIM5	1.021979856	MFAP1
BC014506	-1.007775954			MRPS23
SRD5A2	-2.480267904			PAMR1
UBC	-1.255423842			PER1
MACROD1	-1.093014469			PIR
C11orf54	-1.368737437			PLIN1
HAO2	-2.317018717			PPP1R2
PER1	-1.342771113			PTH1R
CRHBP	-2.591603176			RBP5
LOC553137	-1.855103486			RGPD1
ADH1B	-2.632021297			RHEB
PROZ	-1.877335611			RNF219
SLC28A1	-1.592462371			SLAMF8
CES5A	-1.230058143			SLC16A10
IGFALS	-1.635898926			SLC2A6
GLUD1	-1.053315187			SLC36A1
C9	-2.49334146			SLC45A3
LPAL2	-1.93748657			SORT1
KLF9	-1.173739273			SRC
GPR126	-1.287640568			STEAP4
TMEM27	-1.826657791			STK39
PALM2	-1.453081826			STX6
MDN1	-1.30957122			TESK1
PKLR	-1.571023784			TFRC
CA2	-1.842441672			TMEM184B
MPDZ	-1.226688509			TMEM51
HGFAC	-2.18507121			TPRKB

APOF	-2.283842199	TTC36
TIAM1	-1.225710121	TTYH3
ASPDH	-1.758386436	UBC
CLRN3	-2.03530328	UBD
TTC36	-1.917360665	UTP18
PPAP2B	-1.102162734	VGLL4
IVD	-1.378284419	VIPR1
CPEB3	-1.29517382	WFS1
MPPED1	-1.536167987	YEATS2
SLC38A4	-2.055134825	ZCCHC17
IGSF9	-1.195676797	ZSWIM5
PBLD	-2.073562078	
AFM	-2.289448998	
ALDOB	-2.46915274	
NR1I2	-1.940711069	
RDH5	-1.333966311	
ECM1	-1.435737361	
ZKSCAN1	-1.493857644	
MBL1P	-1.012510202	
ACADSB	-1.697617906	
IL6R	-1.504992642	
LYRM5	-1.013466872	
GLYATL1	-1.975404392	
FOSB	-2.055346461	
SHMT1	-1.517104092	
GLYAT	-2.255110599	
FXYD1	-1.616926949	
ACADL	-1.559484744	
ABAT	-1.832839272	
PDE7B	-1.156122434	
SLC16A10	-1.022747712	
AGL	-1.018815491	
ETFDH	-1.181953912	
SC5DL	-1.4278877	
BC071803	-1.673989436	
BC035094	-1.581689946	
TAT	-2.417766564	
NPPC	-1.018390417	
ERRFI1	-1.871429374	
NAT2	-2.158567609	
DIO3AS	-1.076380486	
GSTZ1	-1.378400165	
BMP5	-1.668094606	
BC034307	-1.982144888	

TM6SF2	-1.392082399
AR	-2.213620555
CAT	-1.50717898
SLC39A5	-1.859205125
MARCO	-1.78278014
BCKDHB	-1.387556089
AK123630	-1.567869075
CYP4A11	-2.279333673
DHRS1	-1.236072981
CR613718	-1.050661835
SMAD6	-1.069651718
XYLB	-1.219132612
SLCO1B3	-2.753525153
ANXA10	-2.342463621
STEAP4	-1.566860544
CYP4A22	-2.453548572
GDF2	-1.401624259
USP43	-1.061273318
CXCL2	-1.954225785
LECT2	-2.360573195
FOS	-1.85370867
DNAJC12	-1.826572413
CHAD	-1.336875983
BC047598	-1.952192306
SLC23A2	-1.035624804
CYP2C9	-2.476163179
GPLD1	-1.944720035
SFXN1	-1.313159983
SLCO1B1	-2.433962634
BHMT	-2.081095664
DMRTA1	-1.614282186
RELN	-1.948506834
HAPLN4	-1.144794247
GYS2	-2.624322085
PCK1	-2.272365082
CYP2A6	-2.300829947
F9	-2.144235752
SLC19A3	-1.398222895
SLC25A25	-1.274232741
G6PC	-2.203966664
PPP1R1A	-1.270424011
SORL1	-1.407493515
DMGDH	-1.920182536
MUC3B	-1.71084238

MAN1A1	-1.123262855
FAM180A	-1.222661883
TMEM192	-1.297925077
ASPG	-1.244155349
RASGEF1B	-1.300328742
CFHR3	-2.311765377
SLC4A4	-1.617708224
PGLYRP2	-1.805325061
UGT2B10	-2.001826958
PAMR1	-1.095922921
CFHR4	-2.467123673
GOT2	-1.092050033
NUDT6	-1.024774022
IL27	-1.25665128
SERINC5	-1.06509564
PNPLA3	-1.383690337
DGAT2	-1.457355551
BRP44L	-1.194527056
RBP5	-1.191459868
MT1F	-1.892936805
SDS	-1.894887957
DUSP1	-1.506440542
ANGPTL6	-1.16211325
RCAN1	-1.319977127
HAAO	-1.235856135
CYP2A13	-2.383707003
HAND2	-1.54615095
EGFR	-1.280765138
MAMDC4	-1.094098819
FBL3B	-1.206644689
ADH6	-2.002984913
SLC22A1	-2.249242237

Supplementary Table S4：GSE54238 Clinical Processing Data Table

ID	title	source_name_ch1	characteristics_ch1.2	characteristics_ch1.3	supplementary_file
GSM1310758	NL1	normal liver	age: 44	gender: M	ftp://ftp.ncbi.nlm.nih.gov/geo/samples/GSM1310nnn/GSM1310758/suppl/GSM1310758_RAM818.pair.gz
GSM1310759	NL2	normal liver	age: 41	gender: M	ftp://ftp.ncbi.nlm.nih.gov/geo/samples/GSM1310nnn/GSM1310759/suppl/GSM1310759_RAM373.pair.gz
GSM1310760	NL3	normal liver	age: 49	gender: M	ftp://ftp.ncbi.nlm.nih.gov/geo/samples/GSM1310nnn/GSM1310760/suppl/GSM1310760_RAM670.pair.gz
GSM1310761	NL4	normal liver	age: 30	gender: M	ftp://ftp.ncbi.nlm.nih.gov/geo/samples/GSM1310nnn/GSM1310761/suppl/GSM1310761_RAM119.pair.gz
GSM1310762	NL5	normal liver	age: 35	gender: M	ftp://ftp.ncbi.nlm.nih.gov/geo/samples/GSM1310nnn/GSM1310762/suppl/GSM1310762_RAM176.pair.gz
GSM1310763	NL6	normal liver	age: 41	gender: M	ftp://ftp.ncbi.nlm.nih.gov/geo/samples/GSM1310nnn/GSM1310763/suppl/GSM1310763_RAM528.pair.gz
GSM1310764	NL7	normal liver	age: 48	gender: M	ftp://ftp.ncbi.nlm.nih.gov/geo/samples/GSM1310nnn/GSM1310764/suppl/GSM1310764_RAM562.pair.gz
GSM1310765	NL8	normal liver	age: 44	gender: M	ftp://ftp.ncbi.nlm.nih.gov/geo/samples/GSM1310nnn/GSM1310765/suppl/GSM1310765_RAM716.pair.gz
GSM1310766	NL9	normal liver	age: 44	gender: M	ftp://ftp.ncbi.nlm.nih.gov/geo/samples/GSM1310nnn/GSM1310766/suppl/GSM1310766_RAM868.pair.gz
GSM1310767	NL10	normal liver	age: 47	gender: M	ftp://ftp.ncbi.nlm.nih.gov/geo/samples/GSM1310nnn/GSM1310767/suppl/GSM1310767_RAM500.pair.gz
GSM1310768	IL1	chronic inflammatory	age: 49	gender: M	ftp://ftp.ncbi.nlm.nih.gov/geo/samples/GSM1310nnn/GSM1310768/suppl/GSM1310768_RAM640.pair.gz

		liver			
GSM1310769	IL2	chronic inflammatory liver	age: 45	gender: M	ftp://ftp.ncbi.nlm.nih.gov/geo/samples/GSM1310nnn/GSM1310769/suppl/GSM1310769_RAM706.pair.gz
GSM1310770	IL3	chronic inflammatory liver	age: 31	gender: M	ftp://ftp.ncbi.nlm.nih.gov/geo/samples/GSM1310nnn/GSM1310770/suppl/GSM1310770_RAM683.pair.gz
GSM1310771	IL4	chronic inflammatory liver	age: 42	gender: M	ftp://ftp.ncbi.nlm.nih.gov/geo/samples/GSM1310nnn/GSM1310771/suppl/GSM1310771_RAM381.pair.gz
GSM1310772	IL5	chronic inflammatory liver	age: 42	gender: M	ftp://ftp.ncbi.nlm.nih.gov/geo/samples/GSM1310nnn/GSM1310772/suppl/GSM1310772_RAM857.pair.gz
GSM1310773	IL6	chronic inflammatory liver	age: 47	gender: M	ftp://ftp.ncbi.nlm.nih.gov/geo/samples/GSM1310nnn/GSM1310773/suppl/GSM1310773_RAM299.pair.gz
GSM1310774	IL7	chronic inflammatory liver	age: 36	gender: M	ftp://ftp.ncbi.nlm.nih.gov/geo/samples/GSM1310nnn/GSM1310774/suppl/GSM1310774_RAM530.pair.gz
GSM1310775	IL8	chronic inflammatory liver	age: 44	gender: M	ftp://ftp.ncbi.nlm.nih.gov/geo/samples/GSM1310nnn/GSM1310775/suppl/GSM1310775_RAM572.pair.gz
GSM1310776	IL9	chronic inflammatory liver	age: 45	gender: M	ftp://ftp.ncbi.nlm.nih.gov/geo/samples/GSM1310nnn/GSM1310776/suppl/GSM1310776_RAM955.pair.gz
GSM1310777	IL10	chronic	age: 38	gender: M	ftp://ftp.ncbi.nlm.nih.gov/geo/samples/GSM1310nnn/GSM1310777/suppl

		inflammatory liver			/GSM1310777_RAM570.pair.gz
GSM1310778	CL1	cirrhotic livers	age: 42	gender: M	ftp://ftp.ncbi.nlm.nih.gov/geo/samples/GSM1310nnn/GSM1310778/suppl/GSM1310778_RAM326.pair.gz
GSM1310779	CL2	cirrhotic livers	age: 58	gender: M	ftp://ftp.ncbi.nlm.nih.gov/geo/samples/GSM1310nnn/GSM1310779/suppl/GSM1310779_RAM017.pair.gz
GSM1310780	CL3	cirrhotic livers	age: 26	gender: M	ftp://ftp.ncbi.nlm.nih.gov/geo/samples/GSM1310nnn/GSM1310780/suppl/GSM1310780_RAM603.pair.gz
GSM1310781	CL4	cirrhotic livers	age: 45	gender: M	ftp://ftp.ncbi.nlm.nih.gov/geo/samples/GSM1310nnn/GSM1310781/suppl/GSM1310781_RAM294.pair.gz
GSM1310782	CL5	cirrhotic livers	age: 40	gender: M	ftp://ftp.ncbi.nlm.nih.gov/geo/samples/GSM1310nnn/GSM1310782/suppl/GSM1310782_RAM166.pair.gz
GSM1310783	CL6	cirrhotic livers	age: 35	gender: M	ftp://ftp.ncbi.nlm.nih.gov/geo/samples/GSM1310nnn/GSM1310783/suppl/GSM1310783_RAM412.pair.gz
GSM1310784	CL7	cirrhotic livers	age: 56	gender: M	ftp://ftp.ncbi.nlm.nih.gov/geo/samples/GSM1310nnn/GSM1310784/suppl/GSM1310784_RAM354.pair.gz
GSM1310785	CL8	cirrhotic livers	age: 26	gender: M	ftp://ftp.ncbi.nlm.nih.gov/geo/samples/GSM1310nnn/GSM1310785/suppl/GSM1310785_RAM359.pair.gz
GSM1310786	CL9	cirrhotic livers	age: 37	gender: M	ftp://ftp.ncbi.nlm.nih.gov/geo/samples/GSM1310nnn/GSM1310786/suppl/GSM1310786_RAM539.pair.gz
GSM1310787	CL10	cirrhotic livers	age: 49	gender: M	ftp://ftp.ncbi.nlm.nih.gov/geo/samples/GSM1310nnn/GSM1310787/suppl/GSM1310787_RAM947.pair.gz
GSM1310788	eHCC1	early HCC	age: 57	gender: M	ftp://ftp.ncbi.nlm.nih.gov/geo/samples/GSM1310nnn/GSM1310788/suppl/GSM1310788_RAM232.pair.gz
GSM1310789	eHCC2	early HCC	age: 58	gender: M	ftp://ftp.ncbi.nlm.nih.gov/geo/samples/GSM1310nnn/GSM1310789/suppl/GSM1310789_RAM607.pair.gz

GSM1310790	eHCC3	early HCC	age: 57	gender: M	ftp://ftp.ncbi.nlm.nih.gov/geo/samples/GSM1310nnn/GSM1310790/suppl/GSM1310790_RAM936.pair.gz
GSM1310791	eHCC4	early HCC	age: 65	gender: M	ftp://ftp.ncbi.nlm.nih.gov/geo/samples/GSM1310nnn/GSM1310791/suppl/GSM1310791_RAM451.pair.gz
GSM1310792	eHCC5	early HCC	age: 51	gender: M	ftp://ftp.ncbi.nlm.nih.gov/geo/samples/GSM1310nnn/GSM1310792/suppl/GSM1310792_RAM581.pair.gz
GSM1310793	eHCC6	early HCC	age: 63	gender: M	ftp://ftp.ncbi.nlm.nih.gov/geo/samples/GSM1310nnn/GSM1310793/suppl/GSM1310793_RAM697.pair.gz
GSM1310794	eHCC7	early HCC	age: 49	gender: M	ftp://ftp.ncbi.nlm.nih.gov/geo/samples/GSM1310nnn/GSM1310794/suppl/GSM1310794_RAM099.pair.gz
GSM1310795	eHCC8	early HCC	age: 59	gender: M	ftp://ftp.ncbi.nlm.nih.gov/geo/samples/GSM1310nnn/GSM1310795/suppl/GSM1310795_RAM315.pair.gz
GSM1310796	eHCC9	early HCC	age: 43	gender: M	ftp://ftp.ncbi.nlm.nih.gov/geo/samples/GSM1310nnn/GSM1310796/suppl/GSM1310796_RAM710.pair.gz
GSM1310797	eHCC10	early HCC	age: 47	gender: M	ftp://ftp.ncbi.nlm.nih.gov/geo/samples/GSM1310nnn/GSM1310797/suppl/GSM1310797_RAM287.pair.gz
GSM1310798	eHCC11	early HCC	age: 59	gender: M	ftp://ftp.ncbi.nlm.nih.gov/geo/samples/GSM1310nnn/GSM1310798/suppl/GSM1310798_RAM987.pair.gz
GSM1310799	eHCC12	early HCC	age: 34	gender: M	ftp://ftp.ncbi.nlm.nih.gov/geo/samples/GSM1310nnn/GSM1310799/suppl/GSM1310799_RAM790.pair.gz
GSM1310800	eHCC13	early HCC	age: 47	gender: M	ftp://ftp.ncbi.nlm.nih.gov/geo/samples/GSM1310nnn/GSM1310800/suppl/GSM1310800_RAM481.pair.gz
GSM1310801	aHCC1	advanced HCC	age: 54	gender: M	ftp://ftp.ncbi.nlm.nih.gov/geo/samples/GSM1310nnn/GSM1310801/suppl/GSM1310801_RAM126.pair.gz
GSM1310802	aHCC2	advanced HCC	age: 54	gender: M	ftp://ftp.ncbi.nlm.nih.gov/geo/samples/GSM1310nnn/GSM1310802/suppl/GSM1310802_RAM320.pair.gz

GSM1310803	aHCC3	advanced HCC	age: 42	gender: M	ftp://ftp.ncbi.nlm.nih.gov/geo/samples/GSM1310nnn/GSM1310803/suppl/GSM1310803_RAM384.pair.gz
GSM1310804	aHCC4	advanced HCC	age: 47	gender: M	ftp://ftp.ncbi.nlm.nih.gov/geo/samples/GSM1310nnn/GSM1310804/suppl/GSM1310804_RAM873.pair.gz
GSM1310805	aHCC5	advanced HCC	age: 65	gender: M	ftp://ftp.ncbi.nlm.nih.gov/geo/samples/GSM1310nnn/GSM1310805/suppl/GSM1310805_RAM449.pair.gz
GSM1310806	aHCC6	advanced HCC	age: 64	gender: M	ftp://ftp.ncbi.nlm.nih.gov/geo/samples/GSM1310nnn/GSM1310806/suppl/GSM1310806_RAM360.pair.gz
GSM1310807	aHCC7	advanced HCC	age: 30	gender: M	ftp://ftp.ncbi.nlm.nih.gov/geo/samples/GSM1310nnn/GSM1310807/suppl/GSM1310807_RAM931.pair.gz
GSM1310808	aHCC8	advanced HCC	age: 55	gender: M	ftp://ftp.ncbi.nlm.nih.gov/geo/samples/GSM1310nnn/GSM1310808/suppl/GSM1310808_RAM758.pair.gz
GSM1310809	aHCC9	advanced HCC	age: 54	gender: M	ftp://ftp.ncbi.nlm.nih.gov/geo/samples/GSM1310nnn/GSM1310809/suppl/GSM1310809_RAM022.pair.gz
GSM1310810	aHCC10	advanced HCC	age: 52	gender: M	ftp://ftp.ncbi.nlm.nih.gov/geo/samples/GSM1310nnn/GSM1310810/suppl/GSM1310810_RAM277.pair.gz
GSM1310811	aHCC11	advanced HCC	age: 46	gender: M	ftp://ftp.ncbi.nlm.nih.gov/geo/samples/GSM1310nnn/GSM1310811/suppl/GSM1310811_RAM441.pair.gz
GSM1310812	aHCC12	advanced HCC	age: 48	gender: M	ftp://ftp.ncbi.nlm.nih.gov/geo/samples/GSM1310nnn/GSM1310812/suppl/GSM1310812_RAM442.pair.gz
GSM1310813	aHCC13	advanced HCC	age: 37	gender: M	ftp://ftp.ncbi.nlm.nih.gov/geo/samples/GSM1310nnn/GSM1310813/suppl/GSM1310813_RAM502.pair.gz

Supplementary Figure S1 Filter cell standards

a Cell filtration quality control **b** After remove batch effect.

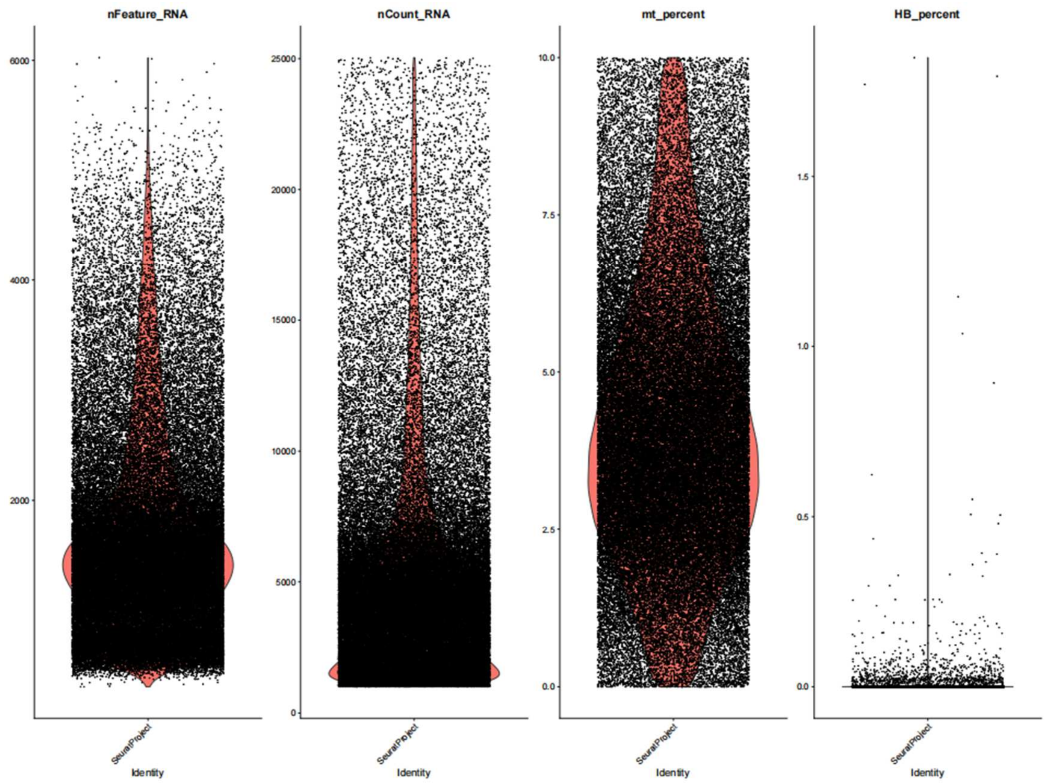

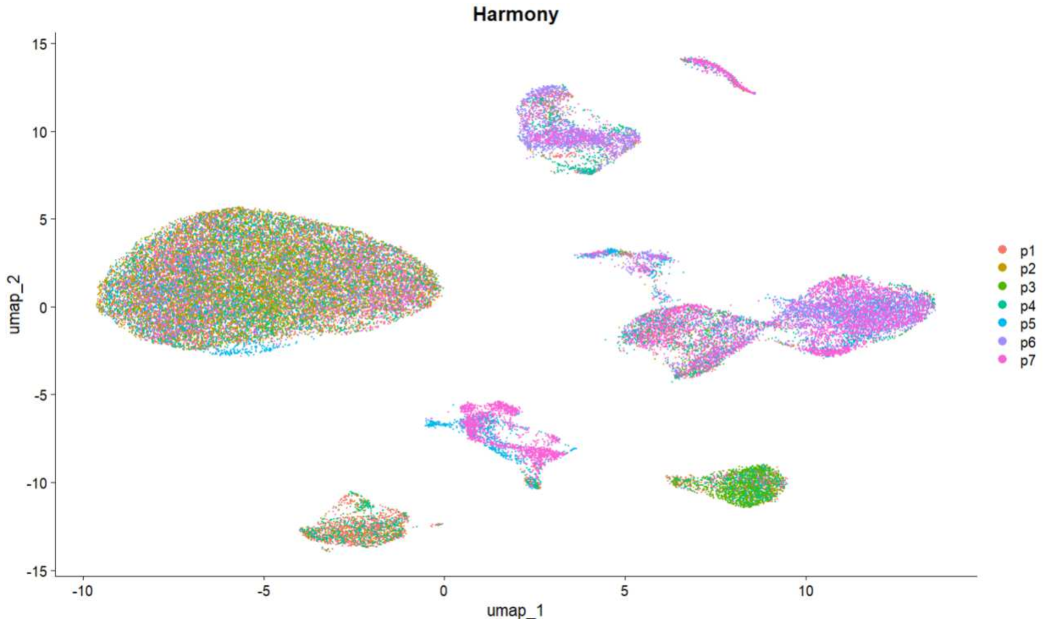

Supplementary Figure S2 Survival analysis.
a Survival curve of SRC **b** Survival curve of FOS

a

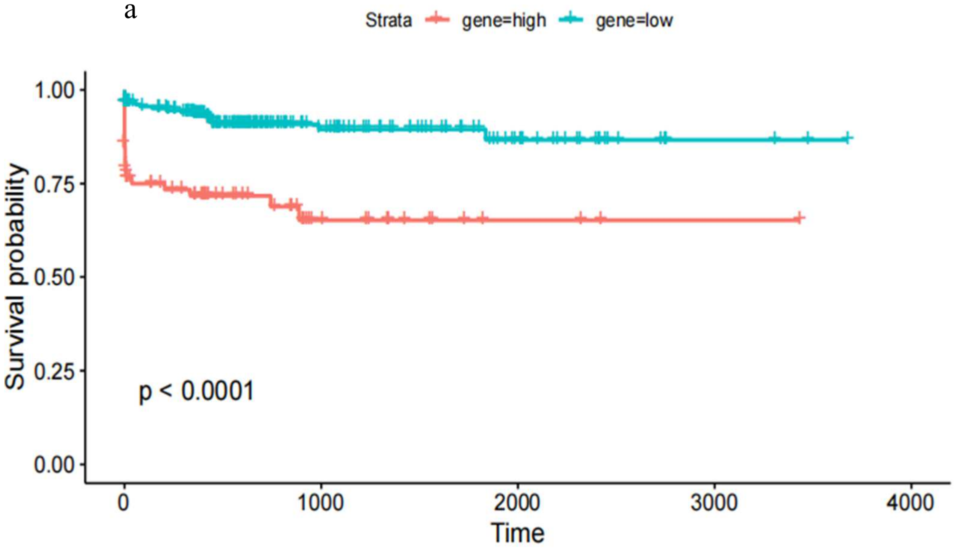

b

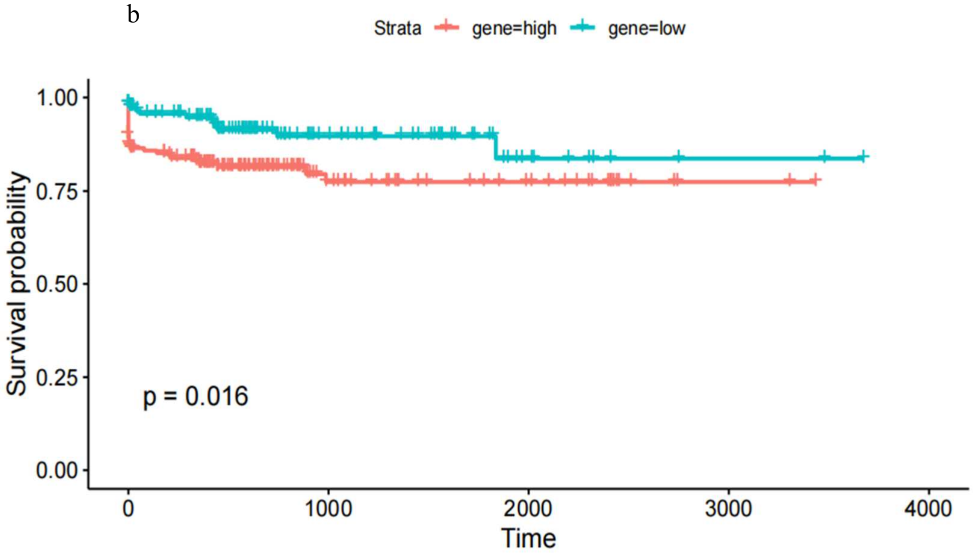

Supplement: Supplementary file 1 [file pharmaceuticals-17-01707-s001.zip › pharmaceuticals-3332073-supplementary.pdf]
